# Inhibition of the DNA damage response phosphatase PPM1D reprograms neutrophils to enhance anti-tumor immune responses

**DOI:** 10.1038/s41467-021-23330-6

**Published:** 2021-06-15

**Authors:** Burhan Uyanik, Anastasia R. Goloudina, Aamir Akbarali, Bogdan B. Grigorash, Alexey V. Petukhov, Sunil Singhal, Evgeniy Eruslanov, Jeanne Chaloyard, Lisa Lagorgette, Tarik Hadi, Ekaterina V. Baidyuk, Hiroyasu Sakai, Lino Tessarollo, Bernhard Ryffel, Sharlyn J. Mazur, Frederic Lirussi, Carmen Garrido, Ettore Appella, Oleg N. Demidov

**Affiliations:** 1 INSERM UMR1231, LipSTIC, University of Burgundy Franche-Comté, Dijon, France; 2 Institute of Cytology, RAS, St. Petersburg, Russia; 3 Laboratory of Cell Biology, Center for Center Research, National Cancer Institute, Bethesda, MD USA; 4 NTU Sirius, Sochi, Russia; 5 Almazov National Medical Research Centre, St. Petersburg, Russia; 6 Department of Surgery, Perelman School of Medicine, University of Pennsylvania, Philadelphia, PA USA; 7 Mouse Cancer Genetics Program, Center for Cancer Research, National Cancer Institute, Frederick, MD USA; 8 INEM, Experimental and Molecular Immunology and Neurogenetics, University of Orléans, CNRS, UMRP735, Orléans, France; 9 PACE, Plateau d’Analyses Chromatographiques et Elémentaires, Department of Pharmacology-Toxicology & Metabolomics, University hospital of Besançon (CHU), 2 Boulevard Fleming, 25030 BESANCON, France; 10 Georges François Leclerc Center, Dijon, France

**Keywords:** Cancer, Cell biology, Immunology, Molecular medicine

## Abstract

*PPM1D*/Wip1 is a negative regulator of the tumor suppressor p53 and is overexpressed in several human solid tumors. Recent reports associate gain-of-function mutations of *PPM1D* in immune cells with worse outcomes for several human cancers. Here we show that mice with genetic knockout of *Ppm1d* or with conditional knockout of *Ppm1d* in the hematopoietic system, in myeloid cells, or in neutrophils all display significantly reduced growth of syngeneic melanoma or lung carcinoma tumors. *Ppm1d* knockout neutrophils infiltrate tumors extensively. Chemical inhibition of Wip1 in human or mouse neutrophils increases anti-tumor phenotypes, p53-dependent expression of co-stimulatory ligands, and proliferation of co-cultured cytotoxic T cells. These results suggest that inhibition of Wip1 in neutrophils enhances immune anti-tumor responses.

## Abstract (web summary)

Wip1 is a negative regulator of the tumor suppressor p53 and is overexpressed in several human cancers. Here the authors show that inactivation of Wip1 in neutrophils promotes p53-dependent expression of co-stimulatory ligands and anti-tumor immune responses, reducing tumor growth in preclinical cancer models.

## Introduction

Immunosurveillance by innate and adaptive immune cells can eliminate aberrant cells and provide effective suppression of tumor initiation^1^. However, sustained interactions between tumors and the immune system may lead to immunosuppression through immunoediting^2^. Immune checkpoint inhibitor-based anticancer therapies employ reducing immunosuppression as an effective therapeutic strategy^3^. However, current anticancer immunotherapies are only fully effective for a fraction of patients, reflecting the complexity of the immune response to tumors^4^. Along with other hematopoietic cells in the tumor microenvironment (TME), neutrophils shape the antitumor immune response and affect the efficacy of immunotherapeutic protocols. Neutrophils belong to the myeloid lineage of the innate immune system and play a dual role in cancer. At the early stages of tumorigenesis, tumor-associated neutrophils (TANs) stimulate antitumor immune responses^5^. Under continuous pathological signaling from tumor cells, neutrophils may acquire immunosuppressive properties and negatively regulate the principal antitumor effector cells, CD8 + cytotoxic lymphocytes, and NK cells^6,7^.

Modulation of immune responses in the TME by p53 is becoming recognized as a key aspect of its tumor-suppressive functions^8–10^. Wild-type p53-induced phosphatase, Wip1, the product of the *PPM1D* gene, is a metal-dependent serine/threonine protein phosphatase that is transcriptionally induced by p53 after exposure to DNA-damaging agents^11,12^. Cell-based studies have demonstrated that Wip1 negatively regulates several tumor suppressors, including p53, ATM, and MAPK^13–16^. The *PPM1D* gene is amplified and/or the Wip1 protein is overexpressed in several human cancers; tumors overexpressing Wip1 often retain wild-type p53, albeit with compromised functionality^17–19^. Wip1 accelerates tumorigenesis in several mouse tumor models and increases the incidence of spontaneous tumors^13,20–23^.

PPM1D exerts cell-type-specific effects during immune cell differentiation^24,25^. It positively regulates T- and B-cell development but negatively regulates neutrophil development^26–30^. Recent work suggests that the level of PPM1D/Wip1 activity in immune cells affects tumor progression. The presence of protein-truncating variants (PTVs) of *PPM1D* in immune cells but not in tumors of breast and ovarian cancer patients was associated with worse outcomes^31^. These somatic PTVs of the *PPM1D* gene were clustered in exon 6 and conferred “gain-of-function” through increased protein stability and activity^32^. For several additional cancers, the presence of PTVs of *PPM1D* in immune cells correlated with worse outcomes for patients^33–36^. Interestingly, in the recently recognized prepathological condition of clonal hematopoiesis of indeterminate potential (CHIP), the same PTVs were found and *PPM1D* was identified as a driver gene^37^. Unlike other CHIP driver genes, *PPM1D* mutations were not associated with increased risk of developing myelodysplastic syndrome (MDS) or acute myeloid leukemia (AML)^38^. Clonal hematopoiesis (CH) resulting from *PPM1D* exon 6 mutations was also found to be significantly associated with increasing age^39^ or prior exposure to chemotherapy^40,41^ and was generally associated with worse outcomes^42^.

We hypothesize that the Wip1 expression level in immune cells affects tumor progression by altering the degree of immunosuppression in the TME. We initiated a study of the effects of Wip1 expression levels or activity on antitumor immune responses.

In this work, we show that *PPM1D* is overexpressed in tumor-infiltrating neutrophils, both in humans and mice, and its genetic deletion or chemical inhibition in myeloid cells increases their anti-tumor phenotypes and suppresses tumorigenesis.

## Results

### Wip1 deficiency in the hematopoietic system suppresses tumor growth

**Fig. 1:**
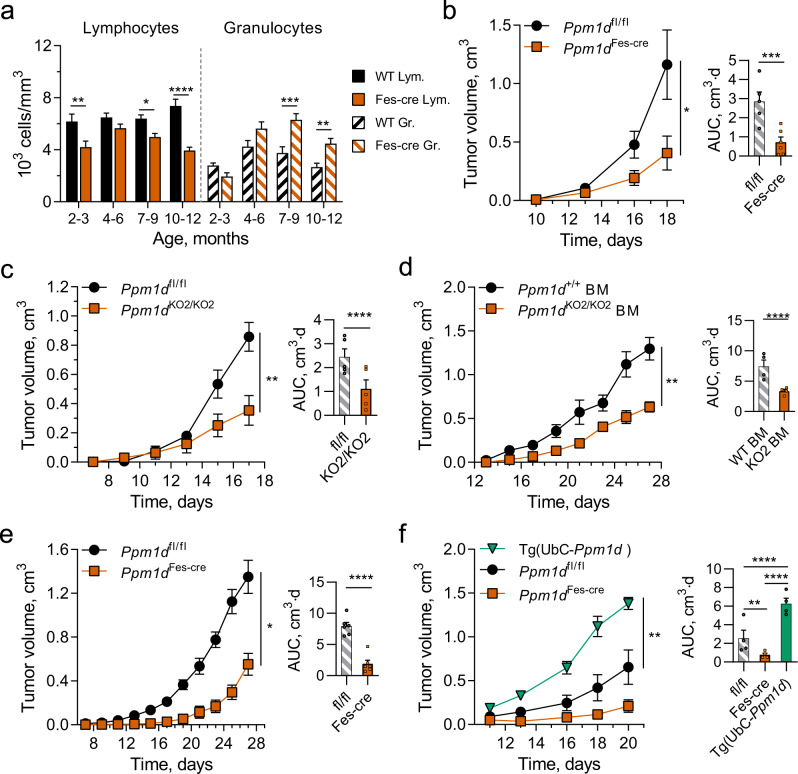
Wip1 deficiency in hematopoietic cells suppresses the growth of solid tumors. **a** Age-stratified lymphocyte and granulocyte numbers in peripheral blood of *Ppm1d*^fl/fl^ and *Ppm1d*^Fes-Cre^ (*Ppm1d*^fl/fl^;Fes-Cre) mice (*n* = 12 each genotype). **b** Tumor volume (left) and tumor growth area under the curve (AUC) (right) for growth of B16 F10 melanoma in *Ppm1d*^fl/fl^ (*n* = 5) and *Ppm1d*^Fes-Cre^ (*n* = 6) mice. **c** Tumor volume (left) and AUC (right) for growth of B16 F10 tumors in *Ppm1d*^fl/fl^ and *Ppm1d*^KO2/KO2^ (*n* = 5 each genotype). **d** Tumor volume (left) and AUC (right) for growth of B16 F10 tumors in lethally irradiated WT mice with subsequent adoptive transfer of WT or *Ppm1d*^KO2/KO2^ bone marrow (BM) cells (*n* = 4 each genotype). **e** Tumor volume (left) and AUC (right) for growth of LLC1 lung carcinoma tumors in *Ppm1d*^fl/fl^ and *Ppm1d*^ΔHSC^ mice (*n* = 6 each genotype). **f** Tumor volume (left) and AUC (right) for growth of LLC1 tumors in *Ppm1d*^fl/fl^, *Ppm1d*^Fes-Cre^, and Tg(UbC-*Ppm1d*) mice (*n* = 4 each genotype). Data are depicted as means ± SEM. Student’s *t* test (two-tailed) (**b**–**e**), ordinary one-way ANOVA (**f**), and ordinary one-way ANOVA with Sidak’s multiple comparison test (**a**): *p < 0.05; **p < 0.01; ***p < 0.001; ****p < 0.0001 (one representative experiment out of 3 is shown for Panel **b**–**f**). Source data are provided as a Source Excel Data file.

To investigate the effects on tumor progression of reducing Wip1 expression in immune cells, we established mouse lines with conditional knockout of the *Ppm1d* gene. Through the Knock-Out Mouse Project (KOMP)^43^, exon 3 of the *Ppm1d* gene was identified as a critical exon, deletion of which results in loss of Wip1 expression and function (Supplementary Fig. 1a). We injected ES cells bearing the *Ppm1d*^Tm1a(KOMP)Wtsi^ gene-trapped allele into pseudopregnant C57Bl/6 mice. Through subsequent crosses of F2 progeny with C57Bl/6 β-actin-cre mice or C57Bl/6 β-actin-flp mice, respectively, we generated mice bearing the *Ppm1d*^Tm1b(KOMP)Wtsi^ allele, a germline *Ppm1d* knockout (referred to herein as *Ppm1d*^KO2^) that expresses the LacZ reporter protein under the control of the endogenous *Ppm1d* promoter, or mice bearing the conditional knockout allele *Ppm1d*^Tm1c(KOMP)Wtsi^ (referred to herein as *Ppm1d*^fl^), which expresses wild-type Wip1. Upon whole-body or tissue-selective exposure to cre recombinase, recombination between LoxP sites flanking exon 3 produces the knockout allele, *Ppm1d*^Tm1d(KOMP)Wtsi^, referred to as *Ppm1d*^KO3^. The human *FES* promoter is highly active in hematopoietic progenitors and myeloid cells; mice expressing the Fes-cre transgene are useful for studying gene deletion in the hematopoietic system^44,45^. Through successive intercrosses of Fes-cre mice with *Ppm1d*^fl/fl^ mice, we produced *Ppm1d*^fl/fl^;Tg(Fes-cre) mice, referred to herein as *Ppm1d*^Fes-cre^ mice, which lack expression of Wip1 in hematopoietic cells (Supplementary Fig. 1b). Analysis of genomic DNA from *Ppm1d*^fl/fl^ mice demonstrated the presence of the floxed exon 3 allele in both skin fibroblasts and liver samples (Supplementary Fig. 1c, left panel). In *Ppm1d*^Fes-cre^ mice, which express cre recombinase from the Fes-cre transgene only in hematopoietic progenitor cells, genomic DNA from liver sample, which contains liver-residing hematopoietic cells as well as hepatic cells, exhibited loss of exon 3 producing the 296-bp product that represented knockout allele (Supplementary Fig. 1c, right panel and Supplementary Fig. 1d). At the same time, skin fibroblasts, which lack hematopoietic cells, demonstrated the absence of the knockout allele in accordance with their respective hematopoietic cell content. Furthermore, we crossed R26R-EYFP reporter mice^46^ with Fes-cre mice to demonstrate successful cre-based recombination occurring in hematopoietic cells but not in other tissue types such as the intestinal epithelium (Supplementary Fig. 1e). Mice homozygous for the floxed allele, *Ppm1d*^fl/fl^, express wild-type Wip1 and are phenotypically indistinguishable from wild-type mice. In agreement with the results described previously for germline Wip1-knockout mice (*Ppm1d*^tm1Lad/tm1Lad^)^27,28,47^, *Ppm1d*^Fes-cre^ mice displayed reduced lymphocyte and increased granulocyte numbers in peripheral blood, with lymphopenia and neutrophilia progressing with age (Fig. 1a).

We investigated the effects of depleting Wip1 in the hematopoietic system on tumor growth using the C57Bl/6 syngeneic B16 F10 melanoma and LLC1 Lewis lung carcinoma tumor models^48,49^. Compared with *Ppm1d*^fl/fl^ mice, we observed significantly reduced rates of B16 F10 tumor growth in *Ppm1d*^Fes-cre^ mice (Fig. 1b). We observed similar reductions in B16 tumor growth in *Ppm1d*^KO2/KO2^ (germline-knockout) mice compared with *Ppm1d*^fl/fl^ mice (Fig. 1c). To test whether the tumor-suppressive characteristic of *Ppm1d*-depleted immune cells is transferable to wild-type mice, we transplanted bone marrow (BM) cells isolated from wild-type or *Ppm1d*^KO2/KO2^ mice into lethally irradiated wild-type mice. We observed reduced rates of B16 tumor growth in WT recipient mice engrafted with BM cells from knockout compared with wild-type mice (Fig. 1d). As deficiency of Wip1 has been reported to impair HSC functionality^50^, we confirmed effective engraftment of YFP-labeled *Ppm1d*^KO2/KO2^ donor cells 12 weeks after transfer into lethally irradiated WT mice (Supplementary Fig. 2). In addition, we challenged *Ppm1d*^fl/fl^ and *Ppm1d*^Fes-cre^ mice with LLC1 Lewis lung carcinoma cells. Similarly, the growth of LLC1 tumors was delayed in *Ppm1d*^Fes-cre^ mice compared with mice expressing wild-type Wip1 (Fig. 1e). Human population studies have demonstrated an association of less favorable patient outcomes with the presence, in blood cells, of *PPM1D* PTV variants that confer increased protein stability^31,36,42^. To investigate the effects of Wip1 overexpression, we used transgenic mice bearing the UbC-*Ppm1d* transgene, which ubiquitously expresses 2- to 4-fold higher levels of Wip1^51,52^. Importantly, the growth of LLC1 carcinoma tumors was significantly faster in UbC-*Ppm1d* transgenic mice compared with either *Ppm1d*^fl/fl^ or *Ppm1d*^Fes-cre^ mice (Fig. 1f). These experiments suggest that high levels of Wip1 in hematopoietic cells increase tumor progression.

### Wip1-deficient neutrophils extensively infiltrate tumors

**Fig. 2:**
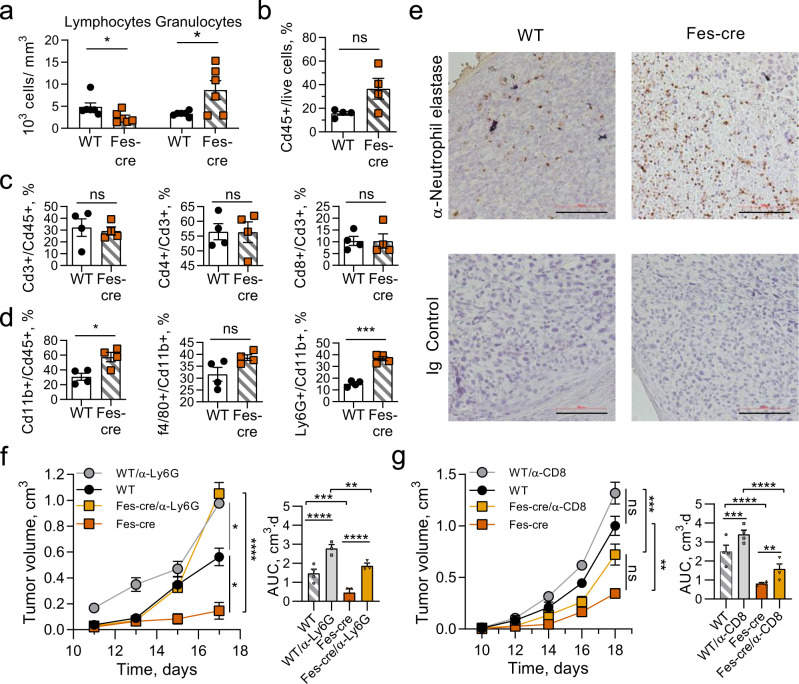
Wip1-deficient neutrophils infiltrate tumors extensively and their depletion accelerates tumorigenesis in Wip1-deficient mice. **a** Peripheral blood composition in *Ppm1d*^fl/fl^ (WT) and *Ppm1d*^Fes-Cre^ (Fes-Cre) mice bearing B16. F10 melanoma tumors (day 16, *n* = 6 each genotype). Panels **b**–**d**: Infiltration of immune cells into B16 tumors in *Ppm1d*^fl/fl^ (WT) and *Ppm1d*^Fes-Cre^ (Fes-Cre) mice (*n* = 4 each genotype). **b** Cd45^+^ leukocytes. **c** Cd3^+^ T cells, Cd4^+^ helper T cells, and Cd8^+^ cytotoxic T cells. **d** Cd11b^+^ myeloid cells, f4/80^+^ macrophages, and Ly6G^+^ neutrophils. **e** Neutrophil infiltration of B16 tumors in *Ppm1d*^fl/fl^ and *Ppm1d*^Fes-Cre^ mice visualized by IHC. Neutrophil elastase-positive cells (α-Elane, brown) with counterstain (blue) (one representative experiment out of 4 is shown). **f**, **g** Tumor volume (left) and AUC (right) for growth of B16 F10 tumors in WT and *Ppm1d*^Fes-Cre^ mice undergoing immune cell depletion by serial injection of neutralizing anti-Ly6G^+^ antibodies (panel **f**, *n* = 4 for WT and *n* = 3 for the remaining groups) or neutralizing anti-CD8^+^ antibodies (panel **g**, *n* = 4 for each group). Data are depicted as means ± SEM. Student’s *t* test (two-tailed) (**a**–**d**) or ordinary one-way ANOVA with Sidak’s multiple-comparison test (**f**, **g**): *p < 0.05; **p < 0.01; ***p < 0.001; ****p < 0.0001, ns—nonsignificant (one representative experiment out of 2 is shown for panels **f** and **g**). Source data are provided as a Source Excel Data file.

Tumor-bearing *Ppm1d*^Fes-cre^ mice exhibited significantly reduced lymphocyte counts and significantly increased granulocyte counts in peripheral blood, compared with tumor-bearing *Ppm1d*^fl/fl^ mice (Fig. 2a). To investigate the mechanisms of tumor suppression resulting from depletion of Wip1 in hematopoietic cells, we characterized immune infiltrates in B16 melanoma tumors in Wip1 WT and Fes-cre mice by flow cytometry (Supplementary Fig. 3). The numbers of CD45 + leukocytes infiltrated into B16–F10 tumors were higher in *Ppm1d*^Fes-cre^mice compared with *Ppm1d*^fl/fl^ mice, but the differences did not reach statistical significance (Fig. 2b). The proportions of CD3 + T cells, CD4 + T cells, or CD8 + T cells (Fig. 2c) did not differ appreciably between *Ppm1d*^Fes-cre^ and *Ppm1d*^fl/fl^ mice, despite substantial peripheral blood lymphopenia. Compared with tumors in WT mice, tumor-immune infiltrates in *Ppm1d*^Fes-cre^ mice exhibited significantly increased proportions of CD11b + myeloid cells, unchanged proportions of F4/80+ tumor-associated macrophages (TAMs), and significantly increased proportions of Ly6G+ tumor-associated neutrophils (TANs) (Fig. 2d). Moreover, immunohistochemical (IHC) analysis revealed substantially increased numbers of neutrophil elastase-positive TANs in tumors from *Ppm1d*^Fes-cre^compared with control mice (Fig. 2e).

To test whether Wip1-deficient neutrophils directly contributed to tumor suppression, we depleted peripheral neutrophils through serial IV injections of neutralizing anti-Ly6G antibodies. Although neutrophil depletion resulted in increased tumor growth in both wild-type and *Ppm1d*^Fes-cre^ mice, neutrophil depletion in *Ppm1d*^Fes-Cre^mice markedly accelerated tumor growth, especially in the later stages (Fig. 2f). Similarly, we depleted peripheral cytotoxic CD8 + T lymphocytes (CTLs) through serial IV injections of neutralizing anti-CD8 antibodies (Fig. 2g). CTL depletion significantly accelerated tumor growth in *Ppm1d*^Fes-cre^mice, but the effects were smaller than the effects of neutrophil depletion. CTL depletion produced a slightly increased endpoint tumor volumes in both genotypes, but the increase did not reach statistical significance. Together, these observations suggest that genetic deletion of *Ppm1d* in immune cells suppressed the growth of isograft tumors in immune-competent mice through increased infiltration of neutrophils.

### Wip1 inhibition affects neutrophil phenotypes

**Fig. 3:**
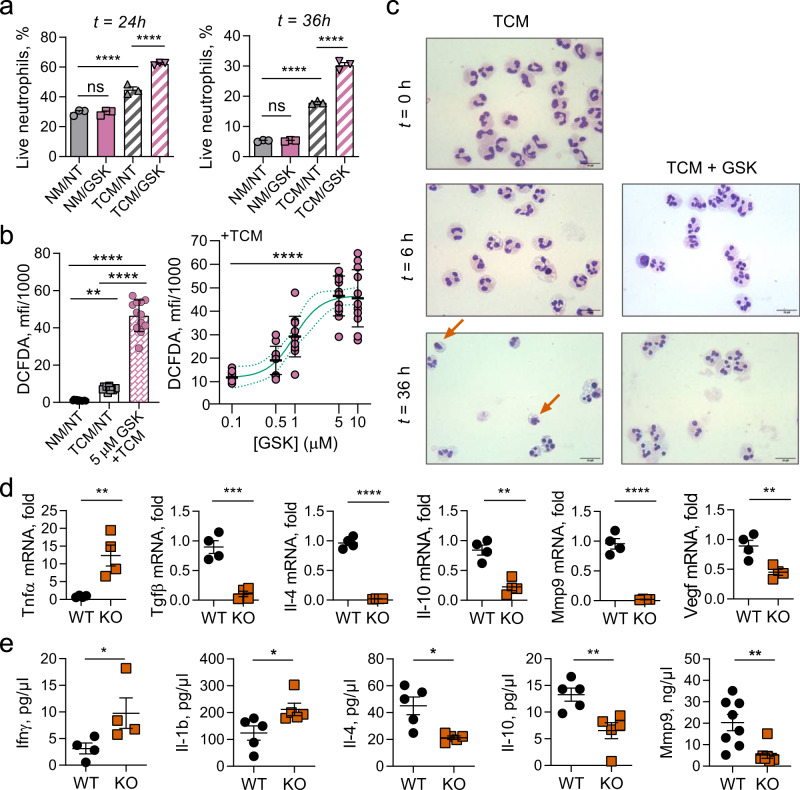
*Ppm1d*-deficient mouse and Wip1-inhibited human neutrophils exhibit characteristics of N1 neutrophils. **a** Survival of human donor blood neutrophils. Freshly isolated neutrophils were cultured in regular media (NA) or in tumor-conditioned media (TCM) and treated with vehicle (NT) or 5 μM GSK2830371 (GSK) for 24 h (left panel) or 36 h (right panel; *n* = 3 for each condition). **b** DFCDA (2′,7′-dichlorofluorescein diacetate) fluorescence as an indicator of reactive oxygen species (ROS) production by donor blood human neutrophils preincubated with vehicle or the indicated concentrations of GSK2830371 for 6 h before activation with PMA (*n* = 12 for each condition). One-way ANOVA with Kruskal–Wallis’ multiple- comparison test, *p* < 0.05; **, *p* < 0.001; ****. **c** Morphology of human donor blood neutrophils cultured in tumor-conditioned medium (TCM) with vehicle (TCM) or 5 μM GSK2830371 (TCM + GSK) for 6 or 36 h. Red arrows indicate apoptotic neutrophils with condensed nuclei. Cytospin spreads were stained with May–Grünwald Giemsa solution and visualized by light microscopy. Scale bar, 20 μm (one representative experiment out of 6 is shown). **d** Relative mRNA levels of various cytokines in B16 melanoma tumors isolated 16 days after engraftment in *Ppm1d* ^+/+^ (WT) or *Ppm1d*^KO2/KO2^ (KO) mice (*n* = 4 for each genotype). **e** Multiplexed bead-based (Luminex) determination of selected cytokine protein levels in B16 tumor lysates from *Ppm1d*^+/+^ (WT) and *Ppm1d*^KO2/KO2^ (KO) mice (*n* = 4 for each genotype for IFN_ϒ_, *n* = 5 for each genotype for Il-1b,4,10, and *n* = 8 for each genotype for MMP9). Data are depicted as means ± SEM. Student’s *t* test (two-tailed) (**d**), Mann–Whitney test (two-tailed) (**e**), or ordinary one-way ANOVA with Sidak’s multiple-comparison test (**a**): *p < 0.05; **p < 0.01; ***p < 0.001; ****p < 0.0001. Source data are provided as a Source Excel Data file.

Recent work has demonstrated that neutrophils are heterogeneous, but the ontogenies and phenotypes of neutrophil subtypes remain incompletely understood^53–55^. A relevant aspect of neutrophil diversity is their potential polarization as antitumor N1 or pro-tumor N2 subtypes^56,57^. We investigated the effects of inhibition of Wip1 phosphatase activity by GSK2830371, a potent and specific inhibitor^58^, or genetic knockout of *Ppm1d* on the characteristics of isolated human and mouse neutrophils. When cultured in normal media, isolated human donor blood neutrophils are short-lived, with only 30% surviving 24 h and 5% surviving 36 h; inhibition of Wip1 phosphatase activity with GSK2830371 did not discernably alter the survival of nonactivated neutrophils (Fig. 3a). Compared with normal media, incubation with tumor-conditioned media (TCM) significantly extended their survival, both at 24 h and 36 h, whereas activation with TCM in the presence of GSK2830371 resulted in further significantly increased survival. Correspondingly, tumor-associated neutrophils (TANs) isolated from B16F10 tumors engrafted in *Ppm1d*^KO2/KO2^ mice compared with *Ppm1d*^+/+^ mice exhibited significantly increased survival during TCM culturing (Supplementary Fig. 4a). Hence, loss of Wip1 activity by chemical inhibition or genetic deletion acts synergistically with soluble factors secreted by tumor cells to extend neutrophil lifespan. Transient production of reactive oxygen species (ROS) through a respiratory burst is a neutrophil effector capability associated with antimicrobial and antitumor activity. Inhibition of Wip1 by GSK2830371 produced a dose-dependent increase in ROS production following PMA stimulation of TCM-activated human donor blood neutrophils (Fig. 3b). Similarly, PMA activation of TCM-stimulated murine BM neutrophils isolated from *Ppm1d*^KO2/KO2^ mice exhibited significantly increased ROS production, compared with similarly treated *Ppm1d*^+/+^ BM neutrophils (Supplementary Fig. 4b), consistent with a previous report^27^. Treatment of WT BM neutrophils with GSK2830371 in TCM prior to PMA stimulation did not significantly increase ROS production. Human neutrophils isolated from healthy donor blood displayed multilobed nuclei characteristic of mature neutrophils; incubation with TCM for 6 h resulted in the prevalence of neutrophils with highly segmented nuclei; addition of GSK2830371 further increased the frequency of highly segmented nuclear morphology (Fig. 3c and Supplementary Fig. 4c). In the absence of GSK2830371, prolonged incubation with TCM resulted in sparse neutrophil survival amid apoptotic neutrophils displaying condensed nuclei, whereas addition of GSK2830371 reduced the incidence of apoptotic cells. In agreement with a previous report^27^, genetic knockout of *Ppm1d* increased the prevalence of neutrophils with highly segmented nuclei (Supplementary Fig. 4d). Hence, genetic ablation of *Ppm1d* or chemical inhibition of Wip1 alters cell-intrinsic characteristics of human or mouse neutrophils consistent with increased N1 polarization.

Through interactions with tumor cells, stromal cells, and other immune cells, neutrophil polarity can influence the cytokine posture of the TME^59,60^. We determined the levels of selected pro- or anti-inflammatory cytokine mRNAs in B16 F10 melanoma tumors isolated from WT or Wip1 KO hosts. Tumors from *Ppm1d*^KO2/KO2^ mice expressed increased levels of TNFα mRNA and reduced levels of Il-4, Il-10, Mmp9, Tgfβ, and Vegf mRNAs, compared with tumors isolated from *Ppm1d*^+/+^ mice (Fig. 3d). TNFα has been shown to induce N1 phenotypes in neutrophils^61^. In contrast, Tgfβ has been implicated in tumor immune suppression and has been shown to induce N2 phenotypes in neutrophils^56^. Compared with N1 neutrophils, N2 neutrophils express higher levels of the cytokines Il-4 and Il-10, the extracellular remodeling factor Mmp9, and the angiogenesis-promoting factor Vegf^62^. We further used a multiplexed bead-based method to quantify the levels of selected cytokines in B16 tumors from *Ppm1d*^+/+^ and *Ppm1d*^KO2/KO2^ mice. The levels of IFNγ and IL-1B were significantly higher and the levels of IL-4, IL-10, and MMP9 were significantly lower in tumors from *Ppm1d*^KO2/KO2^ mice compared with *Ppm1d*^+/+^ mice (Fig. 3e). Exposure to high levels of IFNγ can increase N1 neutrophil polarization^61^.

### Inactivation of Wip1 in neutrophils promotes tumor suppression

**Fig. 4:**
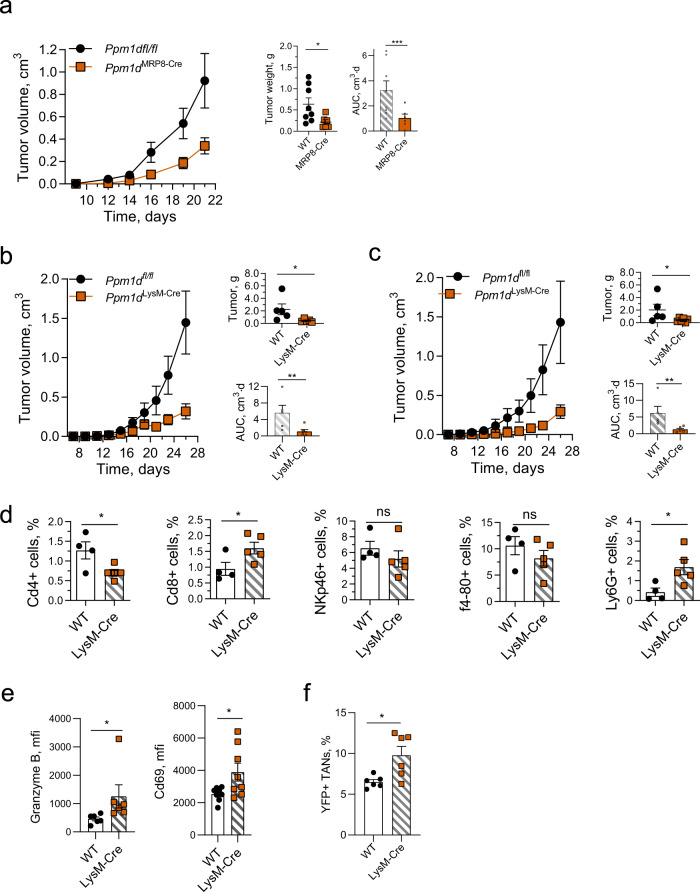
Wip1 deficiency in myeloid immune cells suppresses the growth of solid tumors. **a** Tumor volume (left), endpoint tumor weights (center), and AUC (right) for growth of B16 F10 tumors in *Ppm1d*^fl/fl^ (*n* = 8) and *Ppm1d*^MRP8-CRE^ (*n* = 6) mice. **b** Tumor volume (left), endpoint tumor weights (upper right), and tumor growth area under the curve (AUC) (lower right) for growth of B16 F10 melanoma tumors in *Ppm1d*^fl/fl^ (*n* = 5) and *Ppm1d*^LysM-Cre^ (*n* = 6) mice. **c** Tumor volume (left), endpoint tumor weights (upper right), and AUC (lower right) for growth of LLC1 lung carcinoma tumors in *Ppm1d*^fl/fl^ (*n* = 5) and *Ppm1d*^LysM-Cre^ mice (*n* = 6). **d** Infiltration by immune cell subsets into LLC1 tumors engrafted in *Ppm1d*^fl/fl^ (WT, *n* = 4) and *Ppm1d*^LysM-Cre^ (LysM-Cre, *n* = 5) mice. **e** Expression of activation markers in CD8 + T cells infiltrated in B16 F10 tumors in *Ppm1d*^fl/fl^ (WT) and *Ppm1d*^LysM-Cre^ (LysM-Cre) mice on day 16 (Granzyme B—*n* = 6, Cd69—*n* = 8 each genotype). **f** Infiltration of B16 F10 tumors by YFP + WT^LysM-cre^ or YFP + *Ppm1d*^LysM-cre^ neutrophils after their adoptive transfer in wild-type tumor-bearing mice (*n* = 6 for each genotype). Data are depicted as means ± SEM. Student’s *t* test (two-tailed) (panels **d**, **e** and panels **a**, **b**, **c**: tumor weights) or Mann–Whitney test (two-tailed) (panels **a**, **b**, **e**: AUC, and panel **f**): *p < 0.05; **p < 0.01; ***p < 0.001 (one representative experiment out of 3 is shown for panel **a**, **b** and one out of 2 is shown for panel **f**). Source data are provided as a Source Excel Data file.

To confirm that Wip1-deficient neutrophils suppressed tumor growth in the absence of Wip1 deletion in adaptive immune cells, we generated mouse lines with myeloid targeting of Wip1 knockout. Among available mouse strains, none exhibits exclusive activity in neutrophils; we chose two models, LysM-cre and Mrp8-cre^Tg^, that exhibit high cre activity in neutrophils and differ in their activity profile in other myeloid immune subtypes^63^. In MRP8-cre^Tg^ mice, expression of cre recombinase is driven by the Mrp8 (S100a8) promoter and is highly active in neutrophils and granulocyte–macrophage precursors^64^. We successively crossed *Ppm1d*^fl/fl^ mice with MRP8-cre^Tg^ mice to produce *Ppm1d*^fl/fl^; Tg(S100A8-cre-EGFP)^1Ilw^ mice, referred to herein as *Ppm1d*^MRP8-cre^ mice. *Ppm1d*^MRP8-cre^ mice exhibited significantly reduced growth of B16 melanoma tumors (Fig. 4a). To further test the importance of deletion of Wip1 in myeloid cells, we used a second myeloid-targeting model. In LysM-cre mice, expression of cre recombinase is driven by the lysozyme 2 gene, which is highly active in myeloblasts and their monocyte, macrophage, and granulocyte progeny^63,65^. Through successive intercrosses of LysM-cre mice with *Ppm1d*^fl/fl^ mice, we produced *Ppm1d*^fl/fl^; Lyz2^tm1(cre)Ifo^ mice, referred to herein as *Ppm1d*^LysM-cre^ mice (Supplementary Fig. 5a). We further crossed *Ppm1d*^LysM-cre^ mice with R26R-EYFP reporter mice^46^ to demonstrate genetic deletion of *Ppm1d* (Supplementary Fig. 5b) and loss of Wip1 protein expression (Supplementary Fig. 5c) in Cd11b + , YFP + myeloid cells. Similar to the above results, *Ppm1d*^LysM-cre^ mice exhibited significantly reduced growth of B16 melanoma tumors (Fig. 4b) and LLC1 lung tumors (Fig. 4c), compared with *Ppm1d*^fl/fl^ mice.

We characterized immune infiltrates in LLC1 lung cancer tumors in *Ppm1d*^fl/fl^ and *Ppm1d*^LysM-cre^ mice (Fig. 4d). The numbers of CD4^+^ T cells were significantly reduced and the numbers of CD8^+^ T cells were significantly increased in *Ppm1d*^LysM-cre^ mice compared with *Ppm1d*^fl/fl^ mice, suggesting an increased antitumor engagement of the adaptive immune system. The numbers of NKp46^+^ natural killer cells and of F4–80^+^ macrophages did not differ significantly in tumors from *Ppm1d*^LysM-cre^ mice compared with *Ppm1d*^fl/fl^ mice. Similar to *Ppm1d*^Fes-cre^ mice, the numbers of TANs were approximately fourfold higher in tumors engrafted in *Ppm1d*^LysM-cre^ compared with *Ppm1d*^fl/fl^ mice (*p* = 0.031). Moreover, as indicated by significantly higher expression of Granzyme B and CD69 markers, CD8 + cells infiltrated in B16 F10 tumors in *Ppm1d*^LysM-cre^mice were more highly activated, compared with WT mice (Fig. 4e). We confirmed the increased infiltration of neutrophils in *Ppm1d*^LysM-cre^ mouse tumors by IHC (Supplementary Fig. 5d, e). The chemokine receptor Cxcr2 regulates important aspects of neutrophil behavior, including release from the bone marrow and retention at sites of inflammation, including tumors^66^. Previously, neutrophilia in *Ppm1d*^KO/KO^ mice was attributed to increased neutrophil Cxcr2 expression^27^. We found that the expression levels of Cxcr2 mRNA were significantly higher in *Ppm1d*^KO2/KO2^ TANs infiltrated in B16 F10 tumors, compared with *Ppm1d*^fl/fl^ TANs (Supplementary Fig. 5f). To test whether this is a cell-intrinsic characteristic, we adoptively transferred YFP^+^, *Ppm1d*^+/+^, LysM-cre, or YFP^+^, Ppm1d^fl/fl^, and LysM-cre neutrophils to wild-type mice bearing B16 F10 tumors; YFP + neutrophils with deletion of Wip1- infiltrated tumors in significantly higher numbers compared with wild-type Wip1 YFP + neutrophils (Fig. 4f).

Based on EYFP reporter activity, both the LysM-cre and MRP8-cre models effectively produce deletion in 70–80% of neutrophils^63^. Based on the activity profiles of the LysM-cre and MRP8-cre models, we cannot exclude a possible involvement of Wip1-deficient macrophages in tumor suppression. The possible roles of Wip1-deficient macrophages in the TME lie outside the scope of the current investigation. In combination, the observed substantial increase in tumor infiltration by Wip1-deficient neutrophils, but not Wip1-deficient macrophages (Fig. 2d), and the complete reversal of tumor suppression in Wip1-deficient mice by depletion of neutrophils (Fig. 2f), suggests that Wip1-deficient neutrophils are primarily responsible for the observed phenotype in our models.

### Wip1 inhibition increases expression of 4-1BBL and OX-40L

**Fig. 5:**
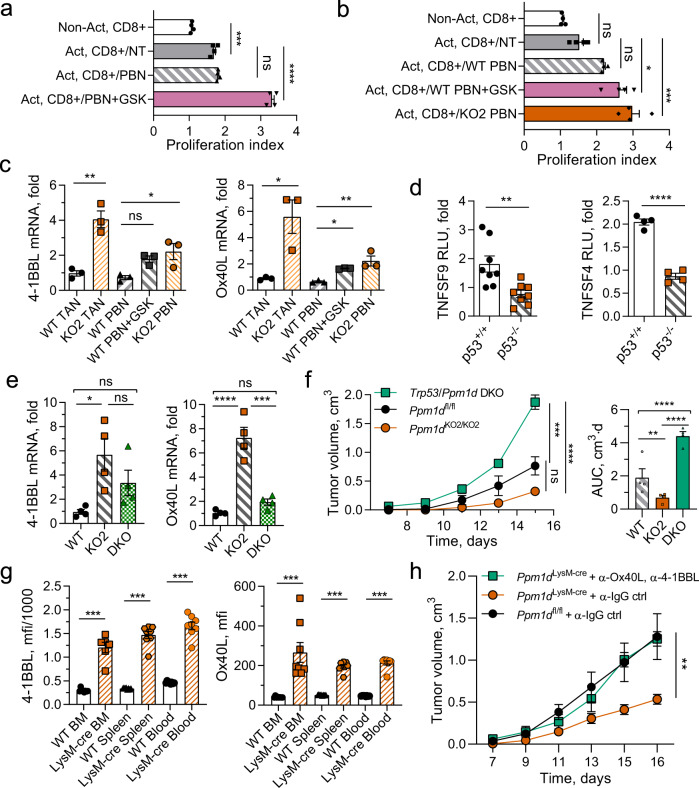
Chemical inhibition of Wip1 or genetic knockout of *Ppm1d* in neutrophils increased cytotoxic T-cell survival through p53-dependent induction of costimulatory ligands. **a** Proliferation of human cytotoxic T lymphocytes. Human donor blood CD8 + lymphocytes were not activated (Non-Act) or activated by incubation with CD3/28 beads (Act.) and cultured alone (NT), or coincubated with isolated human donor blood neutrophils pretreated for 6 h with vehicle (PBN) or 5 μM GSK2830371 (PBN + GSK) (*n* = 4 each condition). **b** Proliferation of murine cytotoxic T lymphocytes. *Ppm1d*^+/+^ peripheral blood Cd8+ lymphocytes were not activated (NT) or activated by incubation on CD3/28-coated plates (Act.) and cultured alone (NT), coincubated with *Ppm1d*^+/+^ neutrophils pretreated for 6 h with vehicle (WT PBN) or 5 μM GSK2830371 (WT PBN + GSK), or coincubated with *Ppm1d*^KO2/KO2^ neutrophils (KO PBN) (*n* = 4 each condition). **c** Expression of 4-1BBL mRNA (left panel) and OX40L mRNA (right panel) in neutrophils isolated from B16 tumors engrafted in *Ppm1d*^+/+^ mice (WT TAN) or *Ppm1d*^KO2/KO2^ mice (KO TAN), isolated from PB of *Ppm1d*^+/+^ mice and treated for with vehicle (WT PBN) or 5 μM GSK2830371 (WT PBN + GSK), or isolated from PB of *Ppm1d* ^KO2/KO2^ mice (KO PBN) (*n* = 3 each condition). **d** Luciferase reporter assay of HCT116 p53^+/+^ and HCT116 p53^−/−^ cells transfected with pGL3 vector expressing luciferase under regulation of the human TNFSF9 (4-1BBL) promoter (left panel, *n* = 8) or the human TNFSF4 (OX40 L) promoter (right panel, *n* = 4). **e** Relative levels of 4-1BBL mRNA (left panel) and OX40L mRNA (right panel) in PBN isolated from *Ppm1d*^+/+^ mice (WT), *Ppm1d*^KO2/KO2^ mice (KO), or *Ppm1d*^KO2/KO2^/*Trp53*^KO/KO^ double-knockout mice (DKO) (*n* = 4 each genotype). **f** Tumor volume (left) and AUC (right) for growth of B16 F10 tumors in *Ppm1d*^fl/fl^ (WT), *Ppm1d*^KO2/KO2^ (KO2), or *Trp53*/*Ppm1d* double-knockout (DKO) mice (*n* = 4 each genotype). **g** Expression of 4-1BBL (left panel) and OX40L (right panel) protein levels on the surface of PPM1D^KO2/KO2^ neutrophils isolated from bone marrow, spleen, and blood (*n* = 8 each, except LysM-Cre BM *n* = 6). **h** Tumor volume for growth of B16 F10 tumors in *Ppm1d*^LysM-Cre^ mice after inactivation of 4-1BBL and OX-40L ligands on the surface of cells with serial injection of neutralizing anti-4-1BBL and anti-OX40L antibodies (*n* = 4 for each group, except *Ppm1d*^fl/fl^ + α-IgG ctrl *n* = 7). Data are depicted as means ± SEM. Student’s unpaired t test (two-tailed) (panel **d**) and Mann–Whitney’s test (two-tailed) (panel **g**), one-way ANOVA (panels **a**–**c**, **e**, **f**), or two-way ANOVA (**h**): *p < 0.05; **p < 0.01; ***p < 0.001, ****p < 0.0001 (one representative experiment out of three is shown for panels **f** and **h**). Source data are provided as a Source Excel Data file.

The activity of cytotoxic CD8 + T cells, the main mediators of antitumor immunity, can be modulated through interactions with neutrophils^59,67^. At early stages of human lung cancer, neutrophils contribute to the antitumor response by stimulating CD8 + T cells through the 4-1BBL/4-1BB and OX-40L/OX-40 pathways^5^. We investigated the effects of chemical inhibition of Wip1 in neutrophils isolated from human donor blood on their ability to costimulate the proliferation of human donor blood T cells (Fig. 5a). CD3/28-activated human CD8 + T cells exhibited significantly increased proliferation compared with nonactivated CD8 + T cells; coculturing with GSK2830371-pretreated human neutrophils further increased proliferation, whereas coculturing with nontreated human neutrophils had no additional effect. In addition, we observed significantly increased proliferation of CD3/28-activated murine CD8 + T cells following coculturing with *Ppm1d*^+/+^ (WT) neutrophils, GSK2830371-treated WT neutrophils, or *Ppm1d*^KO2/KO2^ (KO) neutrophils, compared with activated CD8 + T cells cultured alone (Fig. 5b). Activation of murine CD8 + T cells by itself did not significantly increase their proliferation. Genetic deletion of *Ppm1d* or chemical inhibition of Wip1 activity in neutrophils increased the proliferation of coincubated human or mouse T cells.

Proliferation of CD8 + T cells requires combined signaling through the T-cell receptor (TCR) and costimulatory pathways that include the 4-1BB and OX-40 receptors; the respective ligands, 4-1BBL and OX40 L, are often expressed by antigen-presenting cells and are critical for stimulating the proliferation and activation of cytotoxic T cells^68,69^. We investigated whether expression of these costimulatory ligands in neutrophils was affected by Wip1 phosphatase. The relative levels of 4-1BBL and OX40 L mRNAs were significantly higher in B16 TANs from tumors engrafted in *Ppm1d*^KO2/KO2^ compared with *Ppm1d*^+/+^ (WT) mice (Fig. 5c). For both 4-1BBL and OX-40L, the respective mRNA levels were nearly the same in WT PBNs as in WT TANs, but the levels were higher in KO PBNs or following incubation with GSK2830371. These results suggest that genetic deletion of *Ppm1d* or the loss of Wip1 activity in neutrophils increased the expression of the costimulatory ligands 4-1BBL and OX40L in neutrophils.

Among its many functions, the tumor suppressor p53 regulates immune responses in the tumor microenvironment^9,10^. p53 regulates the expression of several tumor necrosis factor super family (TNFSF) cytokines, including 4-1BBL (TNFSF9) and OX-40L (TNFSF4)^70–72^. We used the public p53 Binding and Expression Resource (BAER)^73^ to examine the correlation between p53 binding to chromatin near the TNFSF9 and TNFSF4 genes and changes in their expression. Chip-seq data for the TNFSF9 gene show a peak of p53 occupancy in chromatin +47 kb from the transcription start site (TSS) in lymphocytes and selected cancer cell lines (Supplementary Fig. 6a). In the corresponding differential gene expression data sets, TNFSF9 was significantly (adjusted p < 0.01) and substantially (fold change >1.5) upregulated in lymphocytes and fibroblasts treated with doxorubicin (DXR) but not in lymphocytes or U2OS cells treated with Nutlin^73,74^. Interestingly, the genomic region +47 kb from the TNFSF9 TSS functions as an enhancer to regulate expression of TNFSF9 and CD70, another TNFSF costimulatory cytokine^75^. In the same datasets, chip-seq data for the TNFSF4 gene show a peak of p53 occupancy in chromatin in intron 1 (+1.3 kb from the TSS or −0.5 kb from an alternative TSS) (Supplementary Fig. 6b). Both regions of high p53 chromatin occupancy contain a sequence resembling the canonical p53 response element (Supplementary Fig. 6c). To provide additional support for p53-dependent induction of the TNFSF9 (4-1BBL) and TNFSF4 (OX-40L) genes, we constructed plasmids expressing luciferase under the control of the TNFSF9 or TNFSF4 promoters and transfected these reporter vectors into HCT116 cells with or without functional p53 (Fig. 5d). With both reporter vectors, we observed significantly increased luciferase activity in HCT116 cells with WT p53 (WT) compared with HCT116 cells lacking p53 (p53^−/−^).

Through intercrosses of mice bearing the *Ppm1d*^KO2^ and *Trp53*^KO^ ^14,15^ knockout alleles, we obtained mice with homozygous double knockout of Wip1 and p53 (*Ppm1d*^KO2/KO2^;*Trp53*^KO/KO^, DKO) and investigated the growth of B16 F10 tumors in *Ppm1d*^fl/fl^ (WT), *Ppm1d*^KO2/KO2^, and DKO mice. The levels of 4-1BBL and OX-40L mRNAs in B16 TANs from *Ppm1d*^KO2/KO2^ mice were significantly increased compared with their respective levels in B16 TANs from WT mice (Fig. 5e). Importantly, the relative mRNA levels for both costimulatory cytokines, although higher, were not significantly increased in B16 TANs from DKO mice compared with B16 TANs from WT mice. We further investigated the rates of B16 melanoma tumor growth WT, KO, and DKO mice (Fig. 5f). In agreement with our earlier results, the growth of B16 F10 tumors in *Ppm1d*^KO2/KO2^ mice was significantly reduced compared with *Ppm1d*^fl/fl^ mice. Deletion of p53 substantially increased the rate of tumor growth compared with either WT or *Ppm1d*^KO2/KO2^. Thus, deletion of p53 attenuated antitumor activity of Wip1-deficient hematopoietic cells both in vitro and in vivo.

In addition, we have verified at protein level, that both ligands 4-1BBL and OX-40L are highly expressed on the surface of *Ppm1d*-deficient neutrophils isolated from bone marrow, spleen, and blood (Fig. 5g). Moreover, the neutralization of 4-1BBL and OX-40L on the surface of cells with specific antibodies in *Ppm1d*^LysM-cre^ mice attenuated the tumor-suppressive effect of *Ppm1d* deletion in myeloid cells and significantly accelerated the tumor growth to the same ratio as in wild-type mice (Fig. 5h).

### Wip1 inactivation in myeloid cells potentiates antitumor therapies

**Fig. 6:**
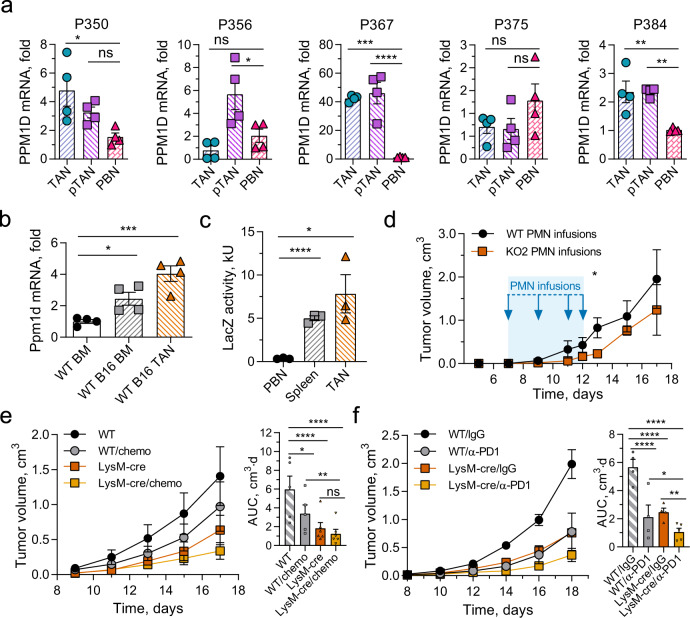
Tumor-associated neutrophils express high levels of Wip1 and inactivation of Wip1 in myeloid cells enhances antitumor responses. **a** Relative PPM1D mRNA levels in neutrophils isolated from surgically resected tumors (TAN), peri-tumor tissues (pTAN), or peripheral blood (PBN) from five patients with Stage I–II lung cancer (*n* = 5 different patients examined over 4 individual experiments). **b** Relative Ppm1d mRNA levels in neutrophils isolated from bone marrow of naive (WT BM) or B16 tumor-bearing (WT BM B16) *Ppm1d*^+/+^ mice and from B16 tumors engrafted in *Ppm1d*^+/+^ mice (WT TAN B16) (*n* = 4 each condition). **c** Flow cytometry analysis of LacZ activity in neutrophils isolated from peripheral blood (PBN), spleen (Spleen), or B16 tumors (TAN) of tumor-bearing *Ppm1d* promoter LacZ reporter mice (*Ppm1d*^+/KO2^) (*n* = 3 each condition). **d** Growth of B16 melanoma tumors in WT mice treated on days 7, 9, 11, and 12 (blue arrows) with injection of neutralizing anti-Ly6G^+^ antibodies to deplete host neutrophils and infusion of *Ppm1d*^+/+^ (WT PMN) or *Ppm1d*^KO2/KO2^ (KO PMN) donor neutrophils (*n* = 4 each genotype). **e** Tumor volume (left) and AUC (right) for growth of B16 F10 tumors in Ppm1d^+/+^ (WT, *n* = 5 each condition) and *Ppm1d*^LysM-Cre^ (LysM-Cre, *n* = 6 each condition) mice without and with oxaliplatin+5FU combination chemotherapy (chemo). **f** Tumor volume (left) and AUC (right) for growth of B16 F10 tumors in Ppm1d^+/+^ (WT, *n* = 4 each condition) and *Ppm1d*^LysM-Cre^ (LysM-Cre, *n* = 4, untreated, *n* = 5 α-PD1) mice without and with anti-PD1 antibody treatment (α-PD1). Data are depicted as means ± SEM. One-way ANOVA with Dunnett’s (**a**–**c**) or Sidak’s (**e**, **f**) multiple- comparison test or Student’s *t* test (two-tailed) (**d**, day 13): *p < 0.05; **p < 0.01; ***p < 0.001; ****p < 0.0001 (one representative experiment out of two is shown for panel **d** and out of three is shown for panel **e**, **f**). Source data are provided as a Source Excel Data file.

Our results show that Wip1 is an important modulator of neutrophil behavior, especially as it relates to the organismal antitumor immune response. In four out of five patients with lung cancer, PPM1D mRNA levels were significantly elevated in either TANs, neutrophils isolated from peritumor tissues (pTANs), or both, compared with the patient’s PBN PPM1D mRNA levels (Fig. 6a). In addition, we investigated the expression level of *Ppm1d* mRNA in neutrophil populations in tumor-free and B16 F10 melanoma tumor-bearing WT mice (Fig. 6b). Compared with *Ppm1d* mRNA levels in BM neutrophils from tumor-free WT mice, *Ppm1d* mRNA levels were significantly increased both in BM neutrophils from tumor-bearing mice and in TANs. Next, we used heterozygous *Ppm1d*^+/KO2^ mice, in which the KO2 allele expresses *Escherichia coli* β-galactosidase (*Lac*Z) under the control of the endogenous *Ppm1d* promoter. Compared with *Ppm1d* promoter activity in PBNs, *Ppm1d* promoter activity was significantly higher in B16 TANs and spleen neutrophils (Fig. 6c).

Our findings suggest that in the tumor microenvironment, Wip1 deficiency reprograms neutrophils toward higher antitumor potential, in part through increased expression of lymphocyte costimulatory ligands. These observations may have potential therapeutic utility. Although the recently developed Wip1 inhibitor is potent and specific, it exhibits poor pharmacokinetics^58^. Moreover, systemic inhibition of Wip1 produces both direct effects of Wip1 inhibition in tumor cells and indirect effects of Wip1 inhibition in infiltrated immune cells. To test whether inhibition of Wip1 activity in neutrophils enhances antitumor responses, we treated tumor-bearing WT mice with serial WT or *Ppm1d*^KO2/KO2^ neutrophil infusions. Mice receiving Wip1-deficient neutrophil infusions showed significantly slower growth of B16 melanoma tumors compared with mice receiving WT neutrophil infusions (Fig. 6d and Supplementary Fig. 7a). Interestingly, tumor growth in mice treated with Wip1-deficient neutrophils accelerated rapidly when treatment was stopped after day 12.

We investigated the potential benefits of combining conventional chemotherapy or current immune checkpoint inhibitor therapy with ablation of Wip1 activity in neutrophils. Treatment with oxaliplatin and 5FU significantly delayed the growth of B16 F10 melanoma tumors in WT mice, but did not significantly delay tumor growth in *Ppm1d*^LysM-cre^mice; combining chemotherapy with Wip1 deficiency in neutrophils resulted in greater tumor suppression (Fig. 6e). Similarly, combining anti-PD1 antibody treatment with Wip1 deficiency in neutrophils more effectively reduced melanoma tumor growth compared with monotherapy with anti-PD1 or Wip1 deficiency alone (Fig. 6f).

## Discussion

Chemotherapy remains one of the main treatment strategies in oncology, but the effects of cytotoxic treatments on the immune system and immune cell–tumor interactions in the TME are currently active areas of investigation. Activation of the DNA damage response pathway by chemotherapeutic drugs not only induces tumor cell lethality, but additionally remodels the TME and alters antitumor immune responses. DNA damage-induced activation of the p53 signaling pathway exerts cell-type- specific selective pressures. In tumors, chemotherapy-induced selection leads to the survival of clones of tumor cells bearing mutations that afford increased resistance to cytotoxic treatments. In the hematopoietic system, chemotherapy-induced selection may lead to CH, in which the presence of somatic mutations of critical genes in hematopoietic progenitors provides survival and proliferative advantages under cytotoxic selection and produces clonal expansion of hematopoietic progeny with altered phenotypes^76^. Among CH driver genes, mutations in *PPM1D* and *TP53* were most significantly associated with prior exposure to chemotherapy^77^. *PPM1D* “gain-of-function” mutations provide selective advantage in tumor cells^32,78,79^ and in the hematopoietic system, resulting in therapy-induced CH^40,80^. Interestingly, the splice variant PPM1D430, which shares salient characteristics with CH-associated *PPM1D* PTVs and has been detected in several cancer cell lines, is expressed only in leukocytes and testes, among normal tissues tested^81^.

The presence of *PPM1D* protein-stabilizing PTVs in the blood cells of patients with several types of solid tumors correlates with worse outcomes^31,33–36,82^ Our study suggests a possible mechanism linking the presence of clonal expansions of blood cells bearing *PPM1D* PTV mutations with worse outcomes for patients with neoplastic disease. Here we have shown that Wip1 negatively regulates the antitumor functions of myeloid cells.

We observed that overexpression of *Ppm1d* in blood cells in mice accelerated the growth of solid tumors, whereas deletion of *Ppm1d* in blood cells suppressed tumor growth. The loss of Wip1 expression in the hematopoietic system significantly increased the infiltration of solid tumors by myeloid granulocytes. Moreover, we observed that the suppression of tumor growth resulting from loss of Wip1 depended on the presence of neutrophils.

*Ppm1d*-knockout mice exhibit marked neutrophilia that progresses with increasing age^28^. Increased *Ppm1d* activity prevents the differentiation of common myeloid progenitors (CMPs) to pro-inflammatory mature granulocytes^27^. We confirmed here that conditional deletion of *Ppm1d* in the hematopoietic system increased the number of neutrophils in mouse blood and altered neutrophil phenotypes.

During the early phase of tumorigenesis, neutrophils contribute to the antitumor immune response, possibly through stimulation of adaptive immunity and enhancement of CD8 + cytotoxic lymphocyte efficiency^5^. During the later stages, tumors evade recognition by the immune system and promote an immune-suppressive state through recruitment of myeloid-derived suppressive cells (MDSCs), including polymorphonuclear (PMN-MDSCs), which share phenotypic and morphologic features with neutrophils^7^. In the TME, MDSCs block the function of the immune effector cells, T-lymphocytes and NK cells, resulting in ineffective removal of tumor cells by the immune system; MDSCs seriously compromise the efficacy of cancer immunotherapies^83^. Recently, strategies directed either to eliminate MDSCs or to reprogram PMN-MDSCs have been proposed to increase the efficiency of immune checkpoint inhibitors^84,85^.

In our study, genetic depletion of *Ppm1d* in neutrophils transformed them into efficient activators of CD8 + cytotoxic lymphocytes, in part, through p53-dependent induction of lymphocyte costimulating ligands 4-1BBL and OX-40L. Due to the limitation of conditional mouse models, we cannot completely exclude the possibility that certain subsets of monocytes/macrophages myeloid cells share some responsibility for the observed phenotype. Though in our model a single *Ppm1d* targeting anticancer strategy was already very efficient, the depletion of *Ppm1d* in myeloid cells potentiated both anticancer chemotherapy and anticancer immunotherapy. Increased CTL activation and survival is critical for efficacy of the chimeric antigen receptor T-cell (CAR-T) transfer and immune checkpoint therapy^86^. There is a rationale for combining immune checkpoint therapy with genetic or chemical inhibition of Wip1. Currently, immune checkpoint therapies target negative regulators of cytotoxic T-lymphocytes and NK cells, such as PD1. In contrast, Wip1 depletion elevates lymphocyte-stimulatory signals in the tumor microenvironment by inducing expression of the stimulatory ligands OX40L and 4-1BBL on the surface of tumor-associated neutrophils. Therefore, by targeting distinct components of the lymphocyte-regulating system, Wip1 inhibition may potentiate anti-PD1 therapy by making antitumor immunotherapy more efficient.

As a practical approach, inhibition of PPM1D activity by chemical compounds in neutrophils or their precursors could be a valuable strategy to increase the efficiency of current anticancer immunotherapies through CTL stimulation in the TME. This strategy will be particularly important in oncological patients with clonal hematopoiesis driven by *PPM1D*-activating mutations to improve treatment outcomes.

## Methods

### Patients

This study was approved by the Institutional Review Boards of the Hospital of the University of Pennsylvania. This study has complied with all relevant ethical regulations and informed consent from participants was obtained. Five patients with stage I–II lung cancer, who were scheduled for surgical resection, consented to tissue collection of a portion of their tumor and/or blood for research purposes. Detailed characteristics of the patients are provided in Supplementary table 1. For donors’s blood, ethical review and approval were done by the French Blood Transfusion Center (Etablissement Français du Sang Bourgogne Franche-Comté, Besançon, France). The blood samples were collected within the agreement between the EFS and the Bourgogne Franche-Comté University according to a national blood bank (Etablissement Français du Sang) rules and policies, with written informed consent specifying the exclusive research purpose and the respect of ethical guidelines.

### Mouse models

All animals were bred and maintained in specific pathogen-free facilities in accordance with FELASA and Animal Experimental Ethics Committee guidelines (University of Burgundy, France) or with NCI Animal Care and Use Committee guidelines (NCI). This study complied with all relevant ethical regulations for animal testing and research and received ethical approval from the Animal Experimental Ethics Committee (University of Burgundy, France) or with the NCI Animal Care and Use Committee (NCI). Animals had water ad libitum and were fed regular chow. All experiments were carried out in accordance with guidelines prescribed by the Ethics Committee at the University of Burgundy and the NCI Animal Care and Use Committee. Experiments were performed in 8–12-week-old female of the immunocompetent C57Bl/6 background. Littermate animals from different cages were randomly assigned into experimental groups and were either cohoused or systematically exposed to other groups’ bedding to ensure equal exposure to common microbiota.

The following strains were generated by our lab using resources from the trans-NIH Knock-Out Mouse Project:^43^ *Ppm1d*^KO2^ (C57BL6-*Ppm1d*^Tm1b(KOMP)Wtsi^), *Ppm1d*^fl^ (C57BL6-*Ppm1d*^Tm1c(KOMP)Wtsi^), and *Ppm1d*^KO3^ (C57BL6-*Ppm1d*^Tm1d(KOMP)Wtsi^). The mouse strains C57BL/6 J, R26R-EYFP (C57BL6-*Gt(ROSA)26Sor*^*tm1(EYFP)Cos*^/J)^46^, *LysM*-cre (B6.129P2-*Lyz2*^tm1(cre)Ifo^/J)^65^, and *MRP8-*cre^Tg^ (B6.Cg-Tg(S100A8-cre-EGFP)^1Ilw^/J)^64^ were purchased from The Jackson Laboratory. *Tp53KO* mice (B6.129-Trp53^tm1Brd^/N)^87^ were provided by L. Donehower (Baylor College of Medicine, Houston, TX, USA). β-actin-cre^Tg^ (C57BL/6J-Tg(β-actin-cre)) mice^88^ and β-actin-flp^Tg^ (C57BL/6J-Tg(β-actin-flp)) mice^89^ were provided by L. Tessarollo (Mouse Cancer Genetics Program, National Cancer Institute, Frederick, MD, USA). *Fes-*cre (C57BL6-Tg(*Fes*-Cre)^31Bsl^) mice^44^ were provided by P. P. Pandolfi (Beth Israel Deaconess Medical Center, Harvard Medical School, Boston, Massachusetts, USA). *Ubc-Ppm1d* C57BL6-Tg(Ubc-Ppm1d) mice^51,52^ were provided by D. Bulavin (Institute for Research on Cancer and Aging Nice, France).

### Cell lines and primary cell cultures

Cell lines were obtained from ATCC and cultured at 37 °C in a humidified 5% CO_2_ atmosphere in Dulbecco’s Modified Eagle’s Medium (DMEM) (LLC1 and HTC116) or in RPMI-1640 Medium (B16 F10 and DLD-1) with 10% (v/v) fetal calf serum supplemented with penicillin–streptomycin/amphotericin B (PSA), and 4 mM of 4-(2-hydroxyethyl)-1-piperazineethanesulfonic acid (HEPES). Cells were regularly tested for mycoplasma contamination. Commonly misidentified cell lines were not used.

Isolated CD3^+^ T cells were cultured in RPMI-1640 medium with 10% (v/v) fetal calf serum (heat-inactivated) supplemented with MEM nonessential amino acids (MEM-NEAA), sodium pyruvate, PSA, and 4 mM HEPES and stimulated with plate-bound antibodies against CD3 (2 µg/ml) and CD28 (2 µg/ml).

### Cancer cell transplantation and organ harvest

B16 F10 murine melanoma cells (2 × 10^5^) (syngenic with C57BL/6 mice) or murine Lewis lung carcinoma (LLC1) cells (3 × 10^5^) (syngenic with C57BL/6 mice) suspended in 100 μl of Dulbecco-modified phosphate-buffered saline (DPBS) were injected subcutaneously into the right flanks of C57BL/6 mice. Tumor growth was monitored every second or third day using electronic calipers. Tumor size was calculated as (tumor volume = (length × width^2^)/2)). The tumor growth Area Under the Curve metric integrates time-series data using trapezoidal approximation^90^. Mice were euthanized when the tumor reached 1000–2000 mm^3^, organs were dissected, and single-cell suspensions were prepared from tumor tissue by mechanical disruption followed by enzymatic digestion of 4–6-mm pieces using a cocktail of Collagenases I (45–60 μ/mL), II (15–20 μ/mL) and IV (45–60 μ/mL), and 100 μg/ml DNase I (Roche) in RPMI medium, as described^5^. Digestion mixtures were sequentially passed through 70-μm and 30-µm cell strainers and washed with PBS supplemented with 2 mM EDTA and 1% FBS. Following lysis of erythrocytes using red blood cell lysis buffer (RBC lysis buffer: 150 mM NH_4_Cl, 10 mM KHCO_3_, and 0.1 mM EDTA), cells were used for immune staining.

### Bone marrow transplantation and chimeric mice

Bone marrow chimeric mice were obtained as described elsewhere^91,92^. Briefly, pooled tibial and femoral bone marrow cells from donor mice were lysed with RBC lysis buffer and assessed for viability with trypan blue. Bone marrow cells were injected retro-orbitally into recipient WT mice (10^7^ cells per each recipient mouse) irradiated with a single dose of 9 Gy. Animals were maintained on trimethoprim–sulfamethoxazole (Hi-Tech Pharmacal) antibiotic water from 1 day prior through 2 weeks after irradiation. Tumor transplantation into chimeric mice was performed at least 8 weeks after reconstitution. Hematopoietic reconstitution of all animals was verified by blood composition analysis using a Scil Vet hemocytometer and by flow cytometry at the end of the experiment.

### Mouse in vivo treatments

Depletion of neutrophils or T cells was affected by intravenous (IV) injection of 200 μg of anti-mouse Ly6G (clone 1A8) or anti-mouse CD8α (clone YTS 169.4) antibodies (Bio X Cell, West Lebanon, NH, USA). Immune cell depletion was verified by blood composition analysis using Scil Vet hemocytometer and flow cytometry analysis (Supplementary Fig. 7c, d). Depleting antibodies were first injected 48 h before tumor isograft implantation; subsequent injections of depleting antibodies were repeated every 3–4 days. BM cells were isolated from WT or *Ppm1d*^KO2/KO2^ mice, and BM PMNs were purified using MACS enrichment following the manufacturer’s protocol, yielding BM PMNs of >90% purity and 95% viability, as determined by flow cytometry (Supplementary Fig. 7a). Mice were transfused with 2 × 10^6^ BM PMNs in 200 µL of physiological serum by retro-orbital injection (at days 7, 9, 11, and 12). For adoptive transfer of YFP + WT^LysM-cre^ and YFP + *Ppm1D*^LysM-cre^ neutrophils, neutrophils were isolated using Neutrophil Isolation Kit (Miltenyi Biotec) and sorted by FACS (ARIA III, BD Bioscience) (Supplementary Fig. 7b); B16 F10 tumor-bearing mice were then transfused 2 days before the endpoint with 2 × 10^6^ cells in 200 µL of physiological serum by retro-orbital injection. For chemotherapy treatment, mice received 5-fluorouracil (5-FU) (5 mg/kg) and oxaliplatin (6 mg/kg) via intraperitoneal injection once weekly (treatment on days 9 and 13). For anti-PD1 treatment, mouse received 200 µg of anti-PD1 (InVivoMab anti-mouse PD1) (Cd279) (clone RMP1–14) via intraperitoneal injection twice weekly (treatment on days 8, 11, 14, and 17). For anti-OX-40L and 4-1BBL treatment, mouse received 250 µg of anti-OX-40L (InVivoMab anti-mouse OX-40L) (Cd134L) (clone RM34L) and anti-4-1BBL (InVivoMab anti-mouse 4-1BBL) (Cd137L) (clone TKS-1) via intraperitoneal injection every 3 days (treatment on days 6, 9, 12, and 15).

### Blood composition analysis

Age-dependent blood composition was analyzed in cohorts of *Ppm1d*^fl/fl^ and *Ppm1d*^ΔHSC^ mice (*n* = 10 each genotype). At intervals, a drop of blood from the tail vein was deposited in an EDTA-coated tube (BD Bioscience). Complete blood counts were performed using an automatic hematoanalyser (ScilVet ABC plus).

### Cell purification and in vitro coculture

#### Mouse

Naive CD3^+^ T cells were obtained from spleens and lymph nodes of C57BL/6 wild-type mice. Cells were purified using the MACS Cell Separation system (Pan T Cell isolation kit, Miltenyi Biotec). Neutrophils were obtained using a mouse Neutrophil Isolation Kit (Miltenyi Biotec). The purity of isolated T-cell and neutrophil populations routinely exceeded 90%. Naive CD3^+^ T cells were stimulated with plate-bound antibodies against CD3 (2 µg/ml) and CD28 (2 µg/ml) (BioLegend) in the absence or presence of neutrophils. B16F10 cells were used as a source of TCM for ex vivo experiments with murine neutrophils.

#### Human

CD3^+^ T cells were obtained from buffy coat preparations of human healthy donor blood. T cells were purified using a Pan T Cell Isolation Kit and restimulated with a human T Cell Activation/Expansion Kit (Miltenyi Biotec). Neutrophils from the same donor were obtained by density-gradient centrifugation. TCM (tumor-conditioned media) was prepared by culturing human melanoma SK-MEL cells in DMEM/10% FBS for 72 h under standard cell culture conditions followed by filtration through a sterile 0.45-μm PVDF filter (Millipore).

#### Preparation of a single-cell suspension from tumor and adjacent lung tissue

Surgically removed fresh lung tumors and adjacent uninvolved lung tissue were processed within 20 min of removal from the patient following the protocol as previously described^5,93^. Briefly, tumor and adjacent uninvolved lung tissue was sliced into 1–2-mm^3^ pieces and digested by enzymes. After 45 min, any visible tumor pieces were vigorously pipetted and then further incubated for 30–50 min under the same conditions. The supernatant was passed through a 70-μM nylon cell strainer (BD Falcon). The remaining pieces in the tube underwent further pipetting before being passed through the same cell strainer. Typically, less than 5% of the tissue remained on the cell strainer. After filtration, the red blood cells were lysed using 1x Red Blood Cell (RBC) Lysis Buffer (Santa Cruz, Dallas, TX). The remaining cells were washed twice in RPMI supplemented with 2% FBS and resuspended in the cell culture media.

#### Neutrophil isolation from human lung tumors

Since temperature gradients can activate neutrophils, all tissues and reagents were maintained at a constant temperature during preparation. After tumor harvest, the neutrophil populations used in this study were prepared at room temperature (RT) and rapidly utilized. TANs were isolated from tumor single-cell suspensions using positive selection of CD66b + cells with microbeads as previously described^5,93^. PBNs were obtained from EDTA anticoagulated peripheral blood collected from lung cancer patients during surgery or from healthy donors. The PBNs were obtained from Lymphoprep (Accu-Prep, 1.077 g/ml, Oslo, Norway) density-gradient centrifugation followed by erythrocyte lysis with 1x RBC Lysis Buffer. To account for any possible effect of tissue digestion enzymes on the function neutrophils, peripheral blood granulocytes were processed in a similar manner. Specifically, peripheral blood granulocytes were incubated with enzymatic cocktail before positive selection using microbeads.

### Flow cytometry

Single-cell suspensions were resuspended in BD stain buffer (BD Bioscience) for 15 min prior to staining with specific antibodies. Antibodies against cell markers: anti-CD45, anti-CD3, anti-CD4, anti-CD8a, anti-CD11b, anti-F4/80, anti-Ly6G, anti-Ly6C, anti-NKp46(CD335), anti-B220, and anti-CD69, were purchased from BD Bioscience and BioLegend; anti-OX40L and anti-41BBL from Miltenyi Biotec (detailed in Supplementary table 4). Samples were mixed with FVS700 (1/7000) and data were acquired on an LSR Fortessa flow cytometer (BD Biosciences) and analyzed with FlowJo software (Tree Star). ROS generation in cultured neutrophils was determined using either DCFDA/H2DCFDA-Cellular ROS Assay Kit (ab113851) following the manufacturer’s protocol or a dihydroethidium (DHE) fluorescent probe. Cells were incubated with DHE (10 μM) in HBSS containing 1.5 mM CaCl_2_ and 1 mM MgCl_2_ for 30 min at 37 °C and analyzed by flow cytometry. LacZ activity was determined by flow cytometry using Fluorescein di[β-D-galactopyranoside (Sigma, F2756) as described elsewhere^94^.

### Immunohistochemistry

FFPE tumor sections (5 µm) from B16–F10 or LLC1 tumors were used to determine the infiltration of neutrophils by immunohistochemistry using rabbit anti-neutrophil elastase antibody (ab68672) (Abcam). Briefly, sections were deparaffinized, then incubated overnight at 4 °C with primary antibodies, washed, and incubated with secondary antibodies (Dako EnVision+ System HRP Labelled Polymer Anti-Rabbit) following the manufacturer’s protocol. Slides were counterstained with hematoxylin counterstain and coverslips were mounted using nonaqueous mounting media.

### Measurement of cytokines

The profiling of selected cytokines and chemokines was performed by a Mouse Luminex assay (R&D systems) according to the manufacturer’s instructions using fresh tumor lysates from equivalent tumor pieces and read on Bio-Plex 200 system (BioRad).

For intracellular cytokine staining, cells were cultured as described above and then stimulated for 4 h at 37 °C in culture medium containing Phorbol 12-myristate 13-acetate (PMA, 50 ng/ml, Sigma) and monensin (2 μM, BioLegend). After staining for surface markers (cf. Flow cytometry section), cells were fixed and permeabilized according to the manufacturer’s instructions (BD Biosciences), then intracellular staining was carried out according to the manufacturer’s protocol using the fixation/permeabilization solution (BD Biosciences).

### Quantitative PCR analysis

Total RNA from T cells was extracted with Trizol (Invitrogen). In all, 300 ng of total RNA was transcribed into cDNA by M-MLV reverse transcriptase with random primers in the presence of RNaseOUT RNAse inhibitor (Invitrogen). cDNAs were quantified by real-time PCR with a SYBR Green Real-time PCR kit (Applied Biosystems) on a Viaa7 detection system (Applied Biosystems, France). Relative mRNA levels were determined with the ΔCt method. Oligonucleotides used for qRT-PCR are described in Supplementary table 2.

### Transient transfections and luciferase transactivation assay

The pTNFSF4-luc and pTNFSF9-luc luciferase reporter constructs were generated by inserting the promoter sequences of the human *TNFSF4* and *TFNS9* genes, respectively, into the multicloning site of the pGL3 basic vector (Promega). Human genomic DNA was isolated from DLD-1 cells by standard methods. Fragments were amplified by high-fidelity PCR using human DNA as the template and specific primers given in Supplementary table 3.

HCT116 (p53^+/+^) or HCT116 p53^−/−^ cells were transiently transfected with reporter plasmids (pTNFSF4-Luc, pTNFSF9-Luc, or pGL3 basic vector) and pSV-β-Galactosidase control vector (Promega) using GenJet™ In Vitro DNA Transfection Reagent (Ver. II) (Sinagen). β-Galactosidase activity was measured using the β-Galactosidase Enzyme Assay System (Promega) after 30 min of incubation at 37 °C with detection at 420 nm. Luciferase activity was measured using the Luciferase Assay System (Promega) according to the manufacturer’s instructions. Firefly luciferase activity was measured using an EnVision 2105 Multimode Plate Reader (PerkinElmer).

### Data collection and analysis

Flow cytometry data were collected using FACSDiva (BD Biosciences, version 8.0.1). qPCR data were collected using ViiA™ 7 Software (Applied Biosystems, version 1.2). Luminescence data were collected using PerkinElmer Envision Manager (v1.13.3009.1401). Flow cytometry data were analyzed on FlowJo software (Tree Star, v10.0.2).

### Quantification and statistical analysis

The results are shown as mean ± SD or SEM, and data sets was compared using unpaired Student’s *t*-test or ordinary one-way or two-way ANOVA as appropriate. We performed statistical calculations with GraphPad Prism 8.3. All *p* values were two-tailed. A *p* < 0.05 was considered statistically significant for all experiments.

### Reporting summary

Further information on research design is available in the Nature Research Reporting Summary linked to this article.

## Supplementary information

Supplementary Information

Reporting Summary

## Data Availability

The p53 ChIP-seq data and linked expression data^73,74^ were obtained from the human p53 Binding And Expression Resource (BAER) data hub [https://orio.niehs.nih.gov/ucscview/nguyen/p53BAER/p53BAER.html] for the human genome assembly hg19 and are available through the UCSC Genome Browser [https://genome.ucsc.edu]. Source data are provided with this paper. The remaining data are available within the paper, Supplementary Information or available from the authors upon request.

## Source data

Source Data
