# Supplementary material for: Inhibition of the DNA damage response phosphatase PPM1D reprograms neutrophils to enhance anti-tumor immune responses
Source: Nat Commun. 2021 Jun 15;12:3622. doi: 10.1038/s41467-021-23330-6 (PMC8206133; doi:10.1038/s41467-021-23330-6)
Supplement: Supplementary file 1 — Supplementary Information [file 41467_2021_23330_MOESM1_ESM.pdf]

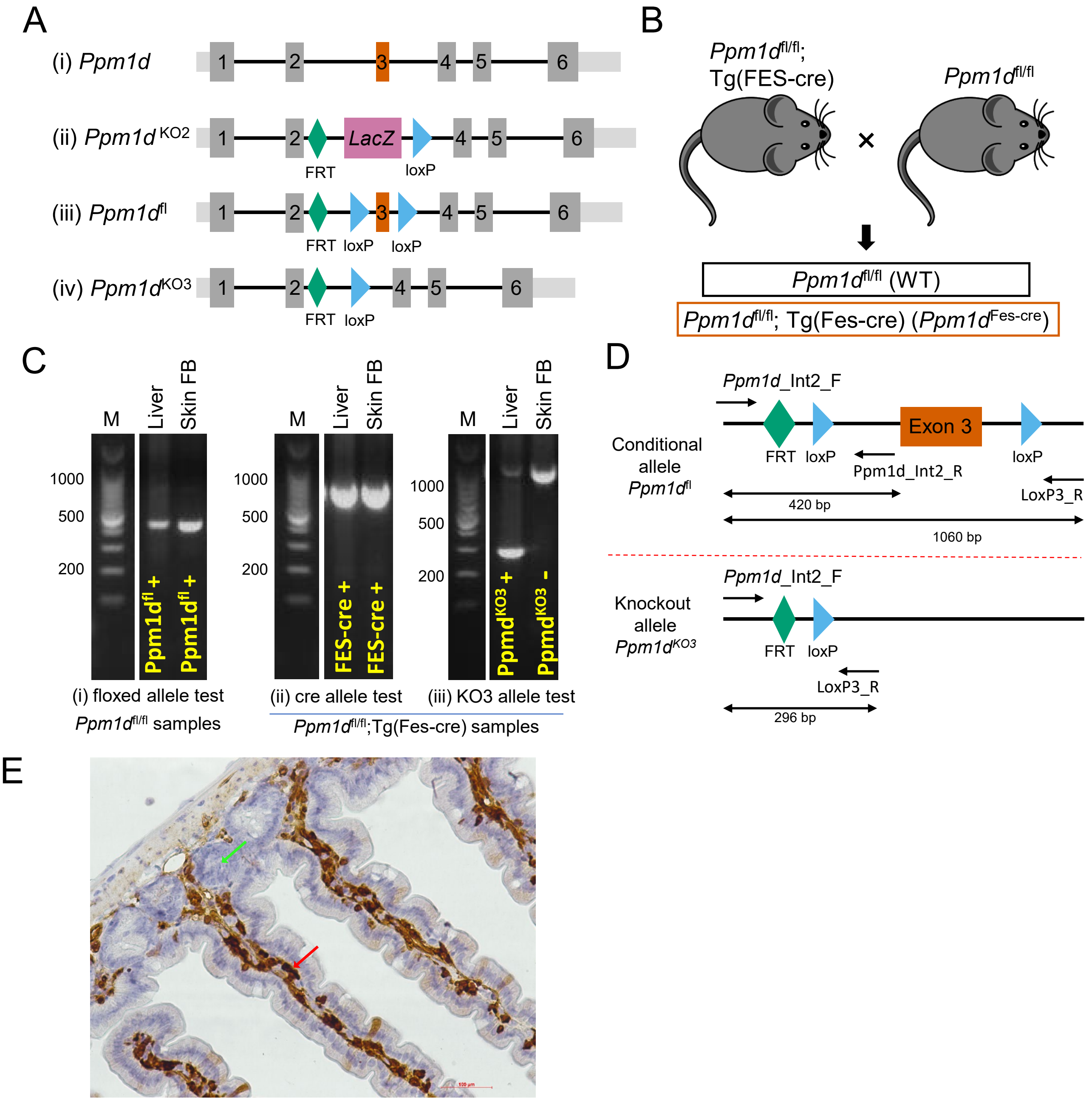

Supplementary Figure 1. Generation of *Ppm1d* conditional knockout mice.

A. Schematic of *Ppm1d* gene and targeted mutations. (i) Wildtype *Ppm1d* allele with critical exon 3 indicated (vermillion); (ii) Knockout allele with targeted LacZ insertion, *Ppm1d*^{KO2} (*Ppm1d*$^{Tm1b(KOMP)Wtsi}$); (iii) Floxed allele with loxP sites flanking exon 3, *Ppm1d*^{fl} (*Ppm1d*$^{Tm1c(KOMP)Wtsi}$), (Conditional allele); (iv) Knockout allele following exposure of cre recombinase, *Ppm1d*^{KO3} (*Ppm1d*$^{Tm1d(KOMP)Wtsi}$), (knockout allele).

B. Breeding scheme for production of littermates with expression of wildtype *Ppm1d* (WT) and with deletion of *Ppm1d* in hematopoietic cells (*Ppm1d*$^{Fes\text{-}cre}$).

C. Genotyping of liver and skin dermal fibroblasts (FB) samples: (i) PCR test for conditional allele (420 bp) in *Ppm1d*$^{fl/fl}$ samples; (ii) PCR test for Fes-cre allele in *Ppm1d*$^{Fes\text{-}cre}$ samples; (iii) PCR test for *Ppm1d*^{KO3} knockout allele (296 bp) in *Ppm1d*$^{Fes\text{-}cre}$ samples. The 1060 bp band corresponds to the unmodified *Ppm1d*^{fl} allele, which is weakly detected in liver and strongly detected in FB samples. (One representative experiment out of 5 is shown).

D. Schematic of PCR strategy for detection of *Ppm1d*^{fl} (conditional, 420 bp) and *Ppm1d*^{KO3} (knockout, 296 bp).

E. Validation of FES-cre mouse model. Crossing of mice bearing the FES-cre transgene with R26R-EYFP reporter mice produced offspring in which FES-driven expression of cre recombinase in hematopoietic stem cells resulted in deletion of the floxed STOP cassette, allowing expression of YFP. IHC analysis of intestinal epithelial sections demonstrates expression of YFP in hematopoietic cells (anti-YFP, brown stain, red arrow), but not in intestinal epithelium cells (blue counterstain, green arrow). Scale bar: 100 μm. (One representative experiment out of 4 is shown).

Supplementary Figure 1

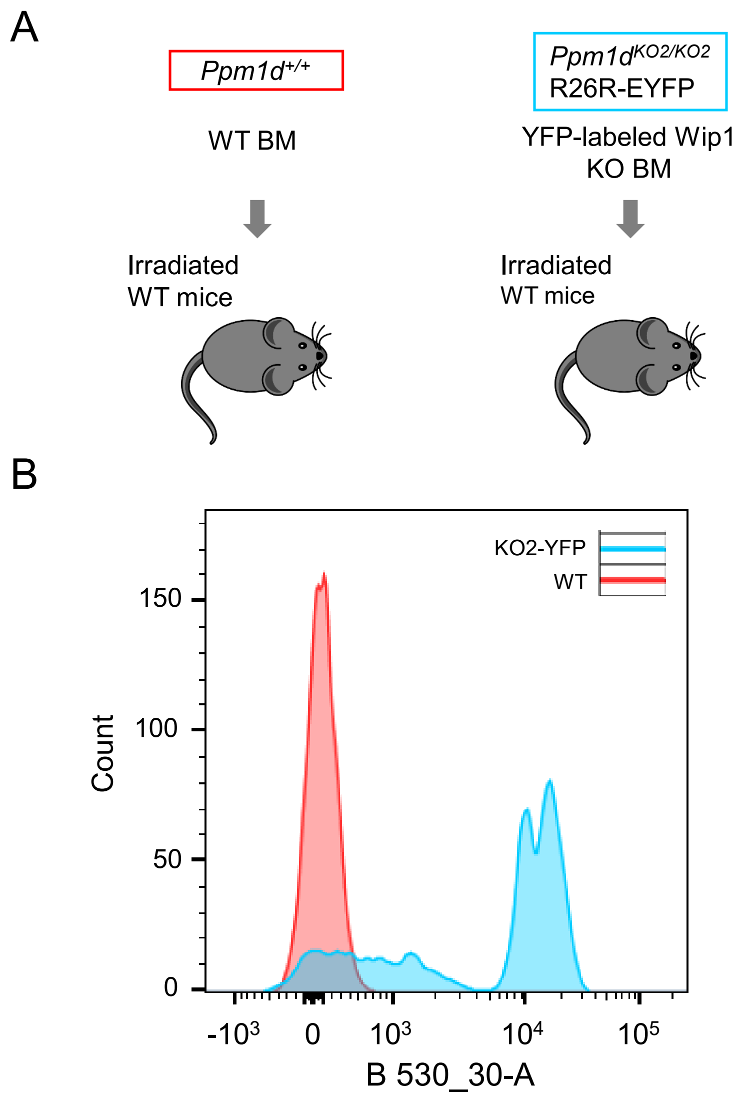

Supplementary Figure 2. Engraftment of Yellow Fluorescent Protein (YFP)-expressing *Ppm1d* knockout bone marrow cells into WT recipient mice.

A. Scheme depicting adoptive transfer of unlabeled WT or YFP-expressing *Ppm1d*$^{KO2/KO2}$ bone marrow (BM) cells into lethally irradiated WT mice.

B. Analysis by flow cytometry of peripheral blood leukocytes 12 weeks after transfer of BM cells from *Ppm1d*$^{+/+}$ or *Ppm1d*$^{KO2/KO2}$; R26R-EYFP mice into lethally irradiated WT mice. Histogram of B530/30 channel shows expression of YFP reporter protein corresponding to transplanted population of BM cells.

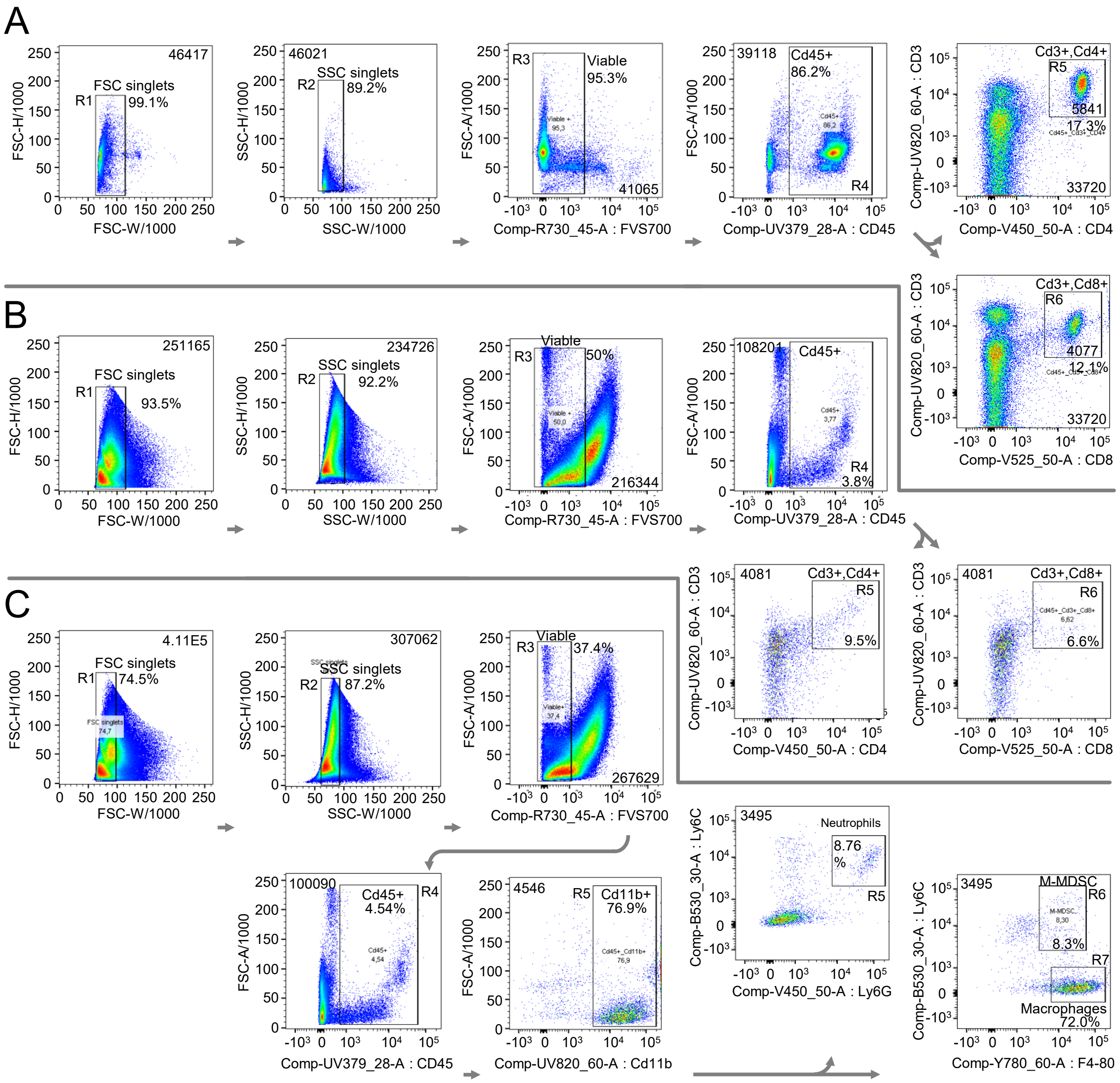

Supplementary Figure 3. Gating strategy for flow cytometry analysis or isolation of immune subsets. Cellular suspensions of selected tissues were analyzed by flow cytometry. Data shown are for WT mice bearing B16 tumors.

A. Gating strategy Cd3+, Cd4+ T cells (Gate R5) and Cd3+, Cd8+ T cells (Gate R6) from spleen.
B. Gating strategy for tumor infiltrated Cd3+, Cd4+ T cells (Gate R5) and Cd3+, Cd8+ T cells (Gate R6).
C. Gating strategy for tumor infiltrated Ly6C+, Ly6G+ Neutrophils (Gate R5), Ly6C+, F4-80+ Monocytic, Myeloid-derived Suppressor cells (M-MDSC) (Gate R6), and Ly6C-, F4-80+ Macrophages (Gate R7).

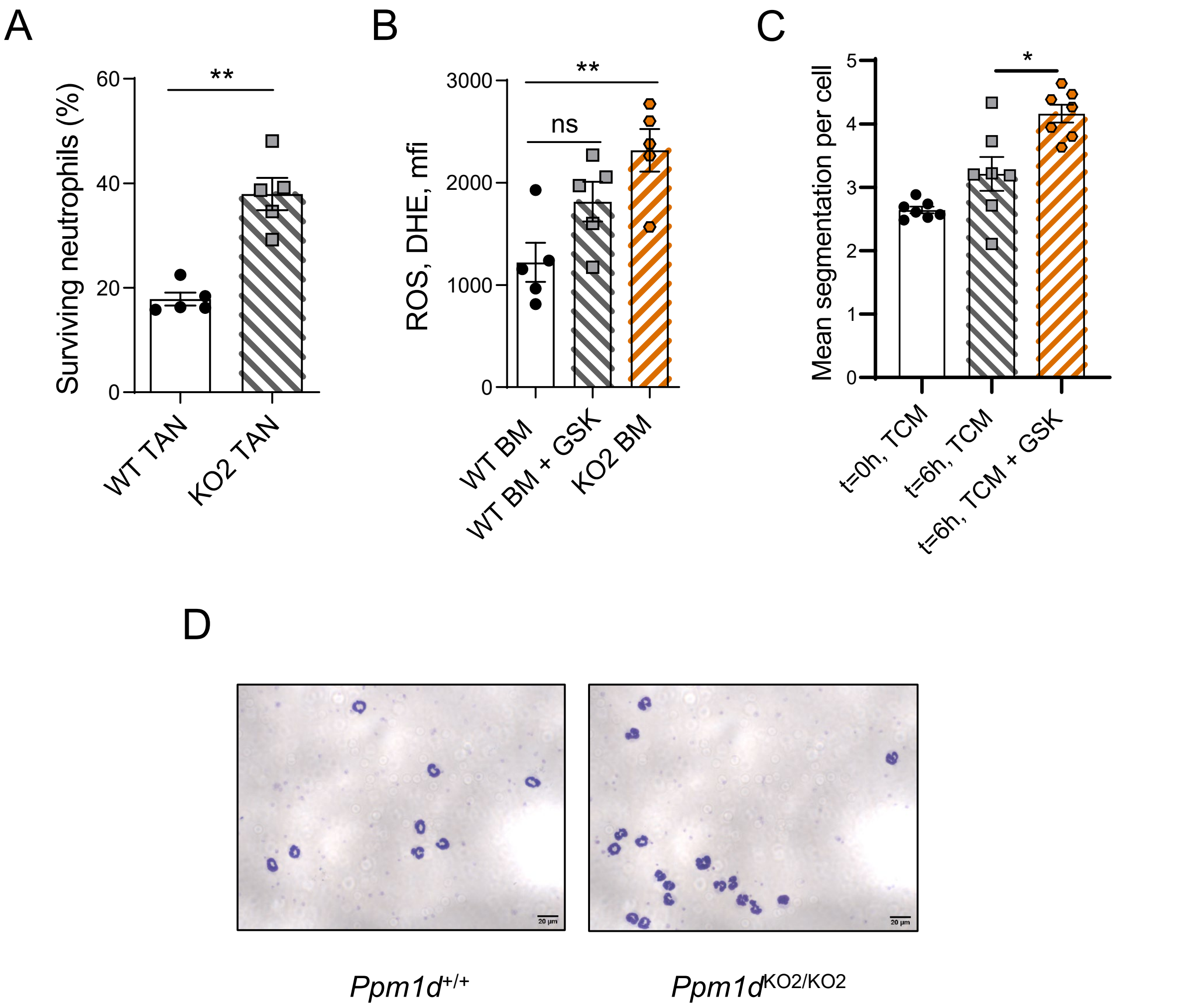

Supplementary Figure 4. Genetic knockout or chemical inhibition of *Ppm1d* affects neutrophil properties.

A. Survival of tumor associated neutrophils (TANs). Neutrophils isolated from B16 F10 tumors engrafted in *Ppm1d*$^{+/+}$ (WT TAN) or *Ppm1d*$^{KO2/KO2}$ (KO2 TAN) mice were cultured for 48 h in tumor conditioned media (TCM). Cell viability was assessed by flow cytometry using 7-aminoactinomycin D (7 AAD). TAN survival: *Ppm1d*$^{+/+}$, 17.8% ± 1.2%, n = 5; *Ppm1d*$^{KO2/KO2}$, 38.0% ± 3.1%, n = 5. Graph displays individual points with mean ± SEM. Statistical significance was estimated by Welch's t test (two-tailed) (**, p < 0.01).

B. Respiratory burst in activated neutrophils. Neutrophils isolated from bone marrow of *Ppm1d*$^{+/+}$ (WT BM) and *Ppm1d*$^{KO2/KO2}$ (KO2 BM) mice were cultured for 6 h in TCM with vehicle or with 5 µM GSK2830371 (+GSK) before activation with PMA. ROS production was detected by dihydroethidium (DHE) fluorescence by flow cytometry, n=5. Graph displays individual points with mean ± SEM . Statistical significance was estimated by one-way ANOVA with Dunnett's multiple testing correction (ns, not significant; **, adj. p < 0.01).

C. Nuclear segmentation of human donor blood neutrophils. Neutrophils isolated from human donor blood were analyzed fresh (t = 0) or cultured for 6 h in TCM without (TCM) or with 5 µM GSK2830371 (TCM + GSK). Cytospin spreads were stained with May-Grünwald Giemsa solution, visualized by light microscopy, and the mean number of nuclear lobes was determined for 50 cells. n = 7 donors for each condition. Graph displays individual points with mean ± SEM. Statistical significance was estimated by Mann Whitney's t test (two-tailed) (*, p < 0.05).

D. Morphology of isolated neutrophils from *Ppm1d*$^{+/+}$ and *Ppm1d*$^{KO2/KO2}$ mice. Neutrophil cytospin spreads were stained with May-Grünwald Giemsa solution and visualized by light microscopy. Scale bar, 20 µm. (One representative experiment out of 4 is shown).

Supplementary Figure 4

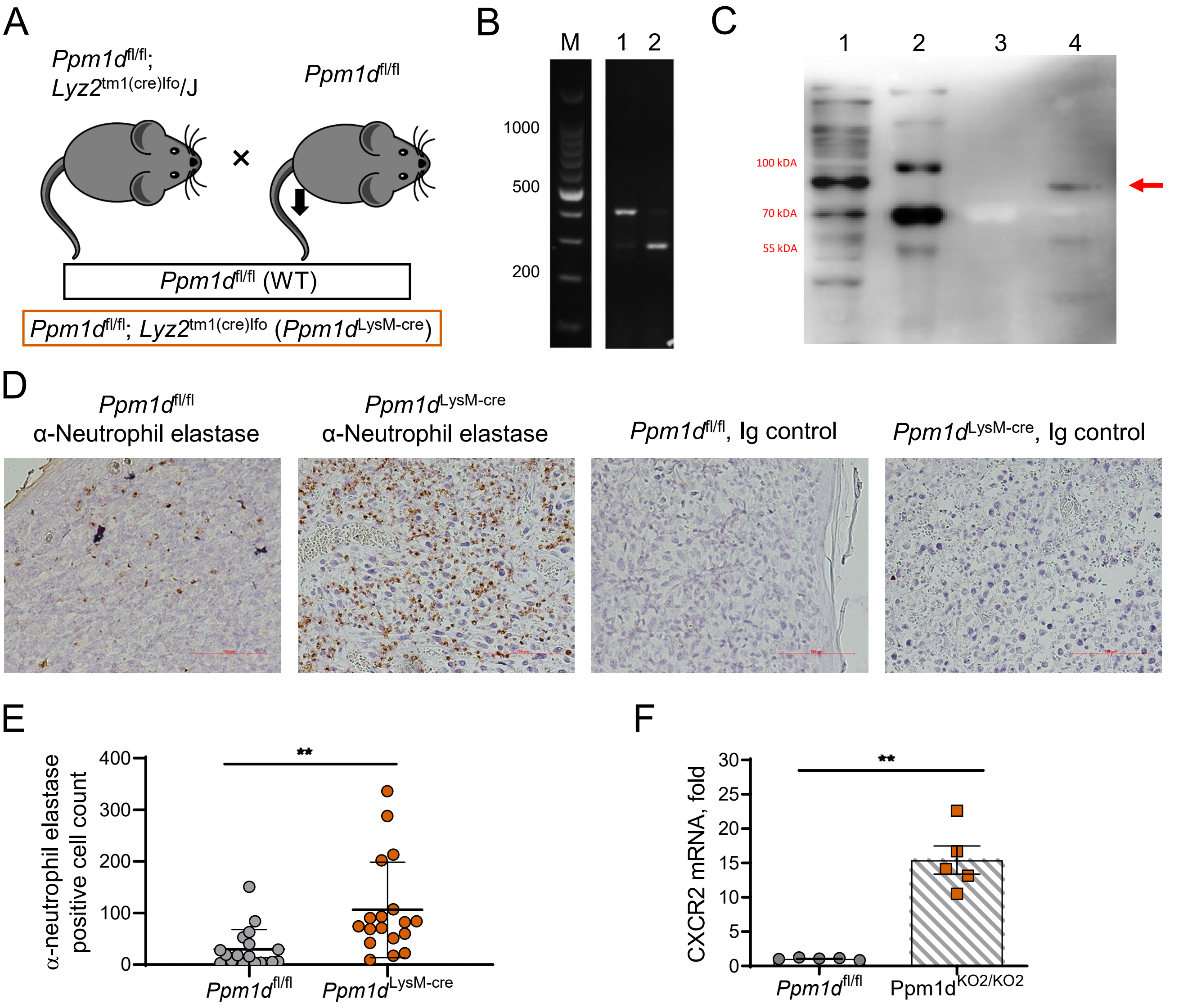

Supplementary Figure 5. Infiltration of LLC1 lung cancer tumors by neutrophils in *Ppm1d*$^{fl/fl}$ and *Ppm1d*$^{LysM\text{-}cre}$ mice.

A. Breeding scheme for production of littermates with expression of wildtype *Ppm1d* (WT) and with deletion of *Ppm1d* in LysM-cre expressing cells and their progeny (*Ppm1d*$^{LysM\text{-}Cre}$).

B. *Ppm1d* locus genotyping of peripheral blood cells sorted by cytometry from *Ppm1d*$^{LysM\text{-}cre}$; R26R-EYFP mice. (1) YFP negative cells displaying *Ppm1d*^{fl} allele band (420 bp); (2) YFP positive cells displaying *Ppm1d*^{KO3} allele band (296 bp). (One representative experiment out of 4 is shown).

C. Validation of the lack of Wip1 expression in YFP-positive myeloid cells from *Ppm1d*$^{LysM\text{-}cre}$ mice. YFP+ and YFP- cells were FACS sorted and Wip1 presence were detected by western blot with anti-Wip1 antibody (D4F7, Cell Signaling). Lanes: 1. HEK293 cells transfected with mouse Wip1; 2. Molecular weight Marker; 3. YFP- myeloid cells; YFP+ myeloid cells. Red arrow indicates Wip1.

D. Neutrophil infiltration into LLC1 tumors in *Ppm1d*$^{fl/fl}$ and *Ppm1d*$^{LysM\text{-}cre}$ mice. FFPE tumor sections (5 µm) were deparaffinized and subjected to immunohistochemistry staining with anti-neutrophil elastase (α-neutrophil elastase, brown) and counterstained with hematoxylin (blue). Scale bars: 100 µm. (One representative experiment out of 4 is shown).

E. Neutrophil elastase positive cell count in engrafted LLC1 tumors. Graph displays individual points with mean ± SD (n = 18, each genotype). Mean neutrophil infiltration is significantly higher (p = 0.0037, Welch's t test (two-tailed)) in LLC1 tumors engrafted in *Ppm1d*$^{LysM\text{-}cre}$ mice, with higher variance (p = 0.0008, F test) compared with *Ppm1d*$^{fl/fl}$ mice.

F. Cxcr2 mRNA expression in wild-type and Wip1-deficient B16 tumor infiltration neutrophils (TANs). Graph displays individual points with mean ± SEM (n = 5, each genotype). Cxcr2 mRNA is significantly higher in Wip1-deficient TANs, p < 0.01, Mann-Whitney test (two-tailed).

A

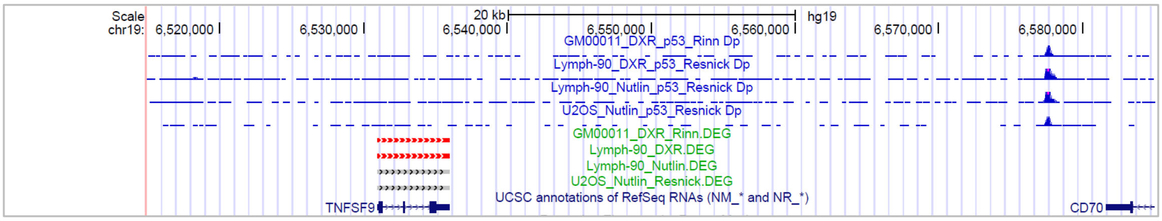

B

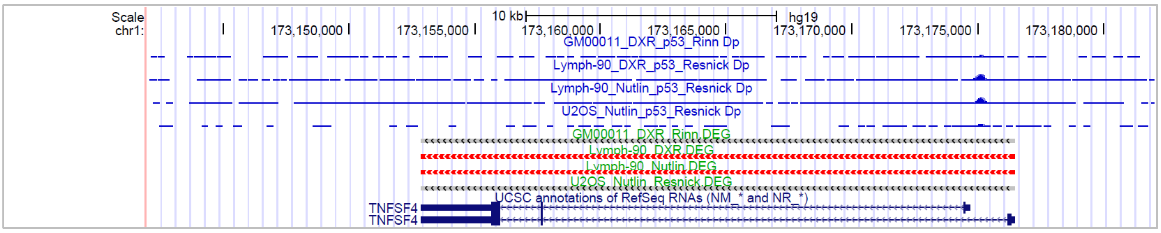

C

Gene	Chr	Str.	Location	RRRCWWGYYYRRRCWWGYYY	p53RE	TSS(kb)
TNFSF9	chr19	(+)	6577605-6577624	GGGCTTGTCTGGGCTTGCCC	20/20	+47
TNFSF4	chr1	(+)	173175146-173175166	AAACAAGCCCAGGCATtTgC	18/20	+1.3/-0.5

Supplementary Figure 6. Binding of p53 to gene regulatory chromatin near 4-1BBL and OX-40L genes. UCSC Genome Browser Graphic on Human Feb. 2009 (GRCh37/hg19) Assembly displaying selected p53 chromatin immunoprecipitation sequencing (ChIP-Seq) tracks and associated differential gene expression tracks from the publicly available p53 Binding and Expression Resource (BAER). Tracks shown: Activated p53 ChIP-seq depth (GM00011_DXR_p53_Rinn, Lymph-90_DXR_p53_Resnick, Lymph 90_Nutlin_p53_Resnick, U2OS_Nutlin_p53_Resnick), Differential gene expression associated with human p53 ChIP-seq datasets (GM00011_DXR_Rinn, Lymph-90_DXR, Lymph-90_Nutlin, U2OS_Nutlin_Resnick (red bar, significantly differentially expressed gene; grey, not significant)), NCBI RefSeq gene predictions.

A. Genomic context of *TNFSF9* (4-1BBL). P53 ChIP-seq peaks are located downstream of the *TNFSF9* gene at a putative enhancer and are associated with *TNFSF9* gene expression changes in some cell lines.
B. Genomic context of *TNFSF4* (OX-40L). P53 ChIP-seq peaks located in intron 1 of the *TNFSF4* gene are associated with *TNFSF9* gene expression changes in some cell lines.
C. Putative p53 binding sites within p53 ChIP-seq peak genomic regions. Mismatches to the consensus sequence are indicated by lower case letters. Also given: p53 RE consensus sequence match score, distance from the transcription start site (TSS).

Supplementary Figure 6

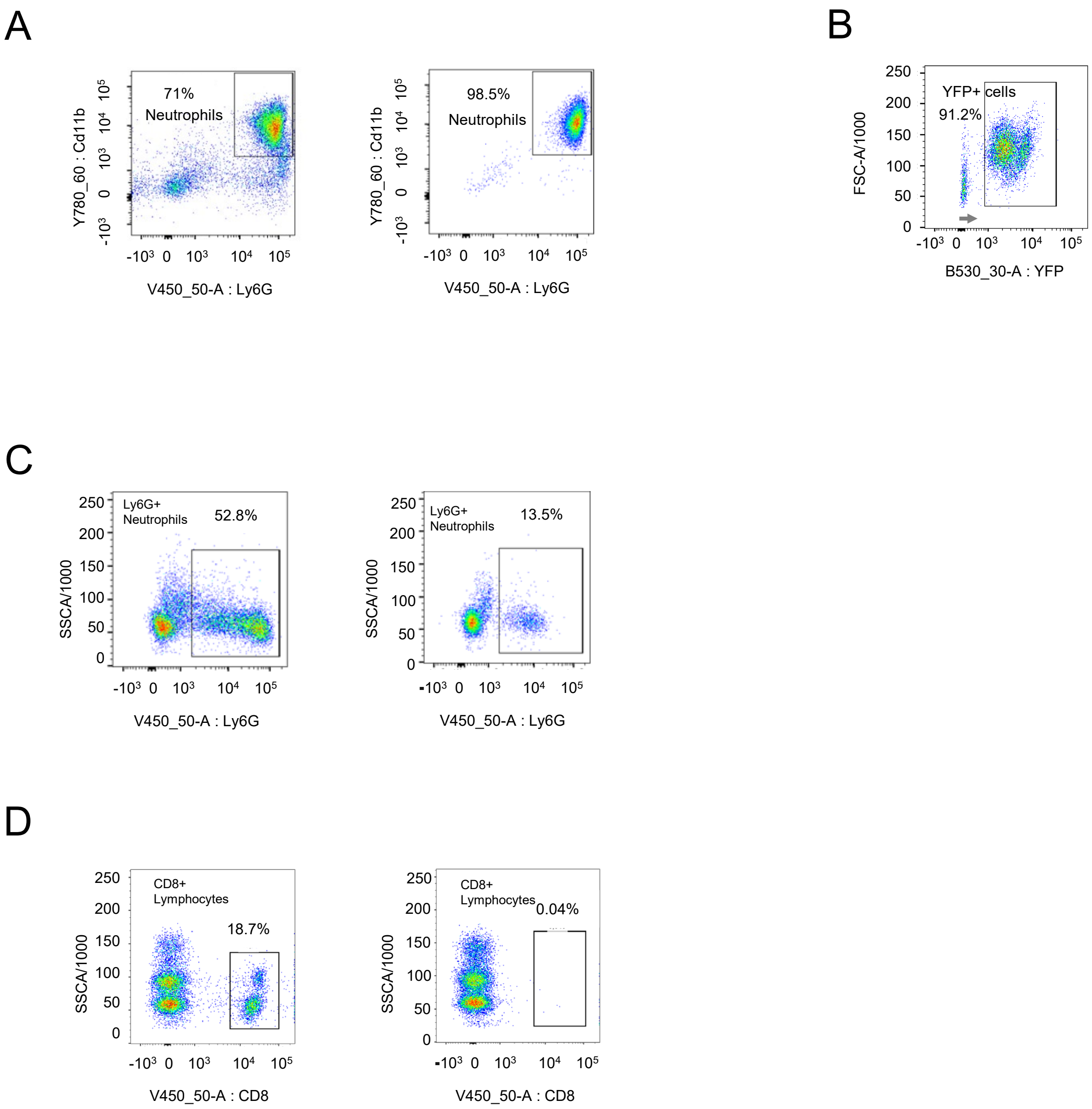

Supplementary Figure 7. Cytometry profile of isolated Immune cells and verification of immune cells depletion efficiency. Cellular suspensions of selected tissues were analyzed by flow cytometry. Data shown are for WT mice bearing B16 tumors.

A. Verification of purity of Miltenyi isolated and FACS sorted YFP+ WT or *Ppm1d*$^{LysM\text{-}cre}$ neutrophils. Left: Neutrophil proportion from cells isolated from BM before Miltenyi sorting (71%); Right: Neutrophil proportion from cells isolated from BM after Miltenyi sorting (98.5%).

B. Verification of purity of Miltenyi isolated and FACS sorted YFP+ WT or *Ppm1d*$^{LysM\text{-}cre}$ neutrophils.

C. Verification of Ly6G+ neutrophils depletion efficiency in mice after treatment with anti-Ly6G antibodies. (Related to Figure 2F). Left: Neutrophil proportion in the blood of non-treated WT mice before depletion (52.8%). Right: Neutrophil proportion in the blood of Ly6G-treated WT mice after depletion (13.5%). FACS analysis of intracellular Ly6G-positive cells.

D. Verification of CD8+ lymphocytes depletion efficiency in mice after treatment with anti-CD8+ antibodies. (Related to Figure 2G). Left: Cd8+ lymphocytes proportion in the blood of non-treated WT mice before depletion (18.7%). Right: Cd8+ lymphocytes proportion in in the blood of CD8-treated WT mice after depletion (0.04%). FACS analysis of CD8-positive cells.

Supplementary Table 1. Patient Characteristics. Related to Figure 6a.

**Cancer Type:**	
Adenocarcinoma	4
Squamous Cell Carcinoma	1
**Age:**	
Median	73
Average	67,4
Range	53-81
**Sex:**	
Male	1
Female	4
**Race:**	
White	4
Black	1
**Tumor Stage:**	
Stage IB	3
Stage IIIA	2
**Tumor grade**	
T1b	1
T2a	4
**Smoking History:**	
Current	1
Never	1
Former	3

Supplementary Table 2. Oligonucleotides for qRT-PCR. Related to Methods.

PRIMER	SEQUENCE (5′ - 3′)	SOURCE
Mouse Ppm1d, FW	GCTAGAGGGAATATCCAGACTGTAGTG	Eurofins Genomics
Mouse Ppm1d, RV	AGTATTTGTTGAATTGGTTGGAATGAGG	Eurofins Genomics
Mouse Tnfsf9, FW	CTGTGTTCCTATCTTCACCC	Eurofins Genomics
Mouse Tnfsf9, RV	TGTCTTCTTCGTACCTCAG	Eurofins Genomics
Mouse Tnfsf4, FW	TGTATGTGTGGGTTCAGCAGCC	Eurofins Genomics
Mouse Tnfsf4, RV	CCCTCAGGAGTCACCAAGGTGGG	Eurofins Genomics
Mouse Gapdh, FW	AATGTGTCCGTCGTGGATCTGA	Eurofins Genomics
Mouse Gapdh, RV	GATGCCTGCTTCACCACCTTCT	Eurofins Genomics
Mouse Hprt1, FW	GCTTGCTGGTGAAAAGGACCTCTCGAAG	Eurofins Genomics
Mouse Hprt1, RV	CCCTGAAGTACTCATTATAGTCAAGGGCAT	Eurofins Genomics
Mouse Il10, FW	TGCTAACCGACTCCTTAATGCAGGAC	Eurofins Genomics
Mouse Il10, RV	CCTTGATTTCTGGGCCATGCTTCTC	Eurofins Genomics
Mouse Il4, FW	AACGTCCTCACAGCAACGAA	Eurofins Genomics
Mouse Il4, RV	CAGCTTATCGATGAATCCAGGCA	Eurofins Genomics
Mouse Mmp9, FW	GCGACCACATCGAACTTCGACACT	Eurofins Genomics
Mouse Mmp9, RV	TCAGGAACTTCCAGTACCAACCGT	Eurofins Genomics
Mouse Tgfb1, FW	AGGTTGGCATTCCATTCAC	Eurofins Genomics
Mouse Tgfb1, RV	AGGGGCCTCTAAGACAGTC	Eurofins Genomics
Mouse Tnfα, FW	GCAAGCTTCGCTCTTCTGTCTACTGAACTT	Eurofins Genomics
Mouse Tnfα, RV	GCTCTAGAATGAGATAGCAAATCGGCTGAC	Eurofins Genomics
Mouse Vegf, FW	GAGCCGAGCTCATGGACG	Eurofins Genomics
Mouse Vegf, RV	TGAACTTGATCACTTCATGGGACT	Eurofins Genomics
Human PPM1D, FW	AGTGATGGACTTTGGAATAT	Eurofins Genomics
Human PPM1D, RV	ACTTCTGGAGAGATGCAGATTAC	Eurofins Genomics
Human GAPDH, FW	AGATCCCTCCAAAATCAAGTGG	Eurofins Genomics
Human GAPDH, RV	GGCAGAGATGATGACCCTTTT	Eurofins Genomics
Human HPRT1, FW	TGAGGATTTGGAAAGGGTGT	Eurofins Genomics
Human HPRT1, RV	GAGCACACAGAGGGCTACAA	Eurofins Genomics

Supplementary Table 3. Oligonucleotides for cloning. Related to Methods

PRIMER	SEQUENCE (5′ - 3′)	IDENTIFIER[1]
Human TNFSF4 promoter FW (with MluI, NheI sites)	CTCTTACGCGTGCTAGCCTGAGACTGAAAGGTCAGC	chr1:173178514-173178534 strand (-)
Human TNFSF4 promoter RV (with XhoI site)	GATCGCAGATCTCGAGCAATCTGGGTAGAGGGAAGAT	chr1:173176321-173176341 strand (+)
Human TNFSF9 promoter FW (with KpnI/Acc65I site)	TTTCTCTATCGATAGGTACCTTTCCGTTCTGCTGGCT	chr19: 6527923-6527942 strand (+)
Human TNFSF9 promoter RV (with HindIII site)	CCGGAATGCCAAGCTTGACGAGAGACTGCGGGAAG	chr19: 6531029-6531048 strand (-)

[1]Human GRCh37/hg19

Supplementary Table 4. Antibody references and dilutions. Related to Methods.

Antibody	Dilution
Rat anti-mouse CD3 molecular complex (clone 17A2) BD Biosciences Cat#741982	1/200
Hamster anti-mouse CD3ε clone 145-2C11), purified BioLegend Cat#100302	2µg/ml
Rat anti-mouse CD4 (clone GK1.5) BD Biosciences Cat#562891; RRID:AB_2737870	1/200
Rat anti-mouse CD8a (clone 53-6.7) BD Biosciences Cat#563068;	1/200
Rat anti-CD11b (clone M1/70) BD Biosciences Cat#741934	1/200
Hamster anti-mouse CD28 (clone 37.51), purified BioLegend Cat#102102	2µg/ml
Rat anti-mouse CD45 (clone 30-F11) BD Biosciences Cat#564279;	1/200
Rat anti-mouse CD45R/B220 (clone RA3-6B2) BD Biosciences Cat#563892	1/200
Hamster anti-mouse CD69 (clone H1.2F3) BD Biosciences Cat#552879	1/100
Rat anti-mouse CD335 (clone 29A1.4) (NK-p46) BD Biosciences Cat#562850	1/200
Rat anti-mouse F4/80 (clone BM8) BioLegend Cat#123114	1/200
Rabbit anti-GFP mAb (clone D5.1) (detects GFP, YFP, CFP) Cell Signaling Technology Cat#2956	1/100
CD137L (4-1BBL) Antibody, anti-mouse, REAfinity (Clone: REA962) Miltenyi Biotec Cat#: 130-116-011	1/100
CD252 (OX40L) Antibody, anti-mouse, APC, REAfinity (Clone: REA960) Miltenyi Biotec Cat#: 130-116-073	1/100
Mouse anti-human/mouse Granzyme B (clone QA16A02) BioLegend Cat#372204	1/100
Rat anti-mouse Ly6C (clone HK1.4) BioLegend Cat#128006	1/100
Rat anti-mouse Ly6G (clone 1A8) BD Biosciences Cat#562737; RRID:AB_2737756	1/100
Rabbit anti-Neutrophil Elastase antibody Abcam Cat#ab68672	1/100
