# Supplementary material for: Inhibition of the DNA damage response phosphatase PPM1D reprograms neutrophils to enhance anti-tumor immune responses
Source: Nat Commun. 2021 Jun 15;12:3622. doi: 10.1038/s41467-021-23330-6 (PMC8206133; doi:10.1038/s41467-021-23330-6)
Supplement: Supplementary file 2 — Reporting Summary [file 41467_2021_23330_MOESM2_ESM.pdf]

nature research

Corresponding author(s): Demidov ON, Appella E, Garrido C

Last updated by author(s): Mar 2, 2021

Reporting Summary

Nature Research wishes to improve the reproducibility of the work that we publish. This form provides structure for consistency and transparency in reporting. For further information on Nature Research policies, see our Editorial Policies and the Editorial Policy Checklist.

Statistics

For all statistical analyses, confirm that the following items are present in the figure legend, table legend, main text, or Methods section.

n/a	Confirmed	
☐	☒	The exact sample size (n) for each experimental group/condition, given as a discrete number and unit of measurement
☐	☒	A statement on whether measurements were taken from distinct samples or whether the same sample was measured repeatedly
☐	☒	The statistical test(s) used AND whether they are one- or two-sided<br>*Only common tests should be described solely by name; describe more complex techniques in the Methods section.*
☒	☐	A description of all covariates tested
☐	☒	A description of any assumptions or corrections, such as tests of normality and adjustment for multiple comparisons
☐	☒	A full description of the statistical parameters including central tendency (e.g. means) or other basic estimates (e.g. regression coefficient) AND variation (e.g. standard deviation) or associated estimates of uncertainty (e.g. confidence intervals)
☐	☒	For null hypothesis testing, the test statistic (e.g. F, t, r) with confidence intervals, effect sizes, degrees of freedom and P value noted<br>*Give P values as exact values whenever suitable.*
☒	☐	For Bayesian analysis, information on the choice of priors and Markov chain Monte Carlo settings
☒	☐	For hierarchical and complex designs, identification of the appropriate level for tests and full reporting of outcomes
☒	☐	Estimates of effect sizes (e.g. Cohen's d, Pearson's r), indicating how they were calculated

Our web collection on statistics for biologists contains articles on many of the points above.

Software and code

Policy information about availability of computer code

Data collection	Flow cytometry data were collected using FACSDiva (BD Biosciences, version 8.0.1). qPCR data were collected using ViiA™ 7 Software (Applied Biosystems, version 1.2). Luminscence data were collected using PerkinElmer Envision Manager (v1.13.3009.1401).
Data analysis	Flow cytometry data were analyzed on FlowJo software (Tree Star, v10.0.2). Statistical analysis was performed using Prism (GraphPad software, version 8.3).

For manuscripts utilizing custom algorithms or software that are central to the research but not yet described in published literature, software must be made available to editors and reviewers. We strongly encourage code deposition in a community repository (e.g. GitHub). See the Nature Research guidelines for submitting code & software for further information.

Data

Policy information about availability of data

All manuscripts must include a data availability statement. This statement should provide the following information, where applicable:

- Accession codes, unique identifiers, or web links for publicly available datasets
- A list of figures that have associated raw data
- A description of any restrictions on data availability

This study did not generate datasets or code. The p53 ChIP-seq data and linked expression data were obtained from the human p53 Binding And Expression Resource (BAER) data hub [https://orio.niehs.nih.gov/ucscview/nguyen/p53BAER/p53BAER.html] for the human genome assembly hg19 and are available through the UCSC Genome Browser [https://genome.ucsc.edu]. Source data are available within the Article, Supplementary Information or available from the authors upon reasonable request.

Field-specific reporting

Please select the one below that is the best fit for your research. If you are not sure, read the appropriate sections before making your selection.

☒ Life sciences ☐ Behavioural & social sciences ☐ Ecological, evolutionary & environmental sciences

For a reference copy of the document with all sections, see nature.com/documents/nr-reporting-summary-flat.pdf

Life sciences study design

All studies must disclose on these points even when the disclosure is negative.

Sample size	No sample-size calculations were performed. Different sample sizes were used depending from the variability and consistency of data between groups and assay sensitivity. Sample sizes for the experiments were chosen based on literature analysis and on investigators' experience with the models.
Data exclusions	No data were excluded from the analysis.
Replication	All findings reported were reproducible and data shown are replicates pooled from >=2 independent experiments, with comparable results in each experiment. The number of replicates are described in each figure legend.
Randomization	Mice were randomly assigned in the different groups. For in vitro experiments, 6–12-week-old mice were matched by age and sex. All transgenic mice used were on a C57BL/6 background and were age-matched with wild-type controls for experiments. For other experiments that did not involve participant groups or animals, no samples randomization were performed as it was not needed.
Blinding	No blinding was performed for the initial testing experiments. The blinding approach was used in experimental repeats whenever possible.

Reporting for specific materials, systems and methods

We require information from authors about some types of materials, experimental systems and methods used in many studies. Here, indicate whether each material, system or method listed is relevant to your study. If you are not sure if a list item applies to your research, read the appropriate section before selecting a response.

Materials & experimental systems

n/a	Involved in the study	
☐	☒	Antibodies
☐	☒	Eukaryotic cell lines
☒	☐	Palaeontology and archaeology
☐	☒	Animals and other organisms
☐	☒	Human research participants
☒	☐	Clinical data
☒	☐	Dual use research of concern

Methods

n/a	Involved in the study	
☒	☐	ChIP-seq
☐	☒	Flow cytometry
☒	☐	MRI-based neuroimaging

Antibodies

Antibodies used	Rat anti-mouse CD3 molecular complex (clone 17A2) BD Biosciences Cat#741982<br>Hamster anti-mouse CD3ε (clone 145-2C11), purified BioLegend Cat#100302<br>Rat anti-mouse CD4 (clone GK1.5) BD Biosciences Cat#562891; RRID:AB_2737870<br>Rat anti-mouse CD8a (clone 53-6.7) BD Biosciences Cat#563068; RRID:AB_2687548<br>InVivoMab anti-mouse CD8a (clone YTS 169.4) BioXCell Cat#BE0117; RRID:AB_10950145<br>Rat anti-CD11b (clone M1/70) BD Biosciences Cat#741934<br>Hamster anti-mouse CD28 (clone 37.51), purified BioLegend Cat#102102<br>Rat anti-mouse CD45 (clone 30-F11) BD Biosciences Cat#564279; RRID:AB_2651134<br>Rat anti-mouse CD45R/B220 (clone RA3-6B2) BD Biosciences Cat#563892<br>Hamster anti-mouse CD69 (clone H1.2F3) BD Biosciences Cat#552879<br>Rat anti-mouse CD335(αNK-p46) (clone 29A1.4) BD Biosciences Cat#562850<br>Rat anti-mouse F4/80 (clone BM8) BioLegend Cat#123114<br>Rabbit anti-GFP mAb (clone D5.1) (detects GFP, YFP, CFP) Cell Signaling Technology Cat#2956<br>CD137L (4-1BBL) Antibody, anti-mouse, REAfinity (Clone: REA962) Miltenyi Biotec Cat#: 130-116-011<br>CD252 (OX40L) Antibody, anti-mouse, APC, REAfinity (Clone: REA960) Miltenyi Biotec Cat#: 130-116-073<br>Mouse anti-human/mouse Granzyme B (clone QA16A02) BioLegend Cat#372204<br>Rat anti-mouse Ly6C (clone HK1.4) BioLegend Cat#128006<br>Rat anti-mouse Ly6G (clone 1A8) BD Biosciences Cat#562737; RRID:AB_2737756

InVivoMab anti-mouse Ly6G (clone 1A8) BioXCell Cat#BE0075-1; RRID:AB_1107721
Rabbit anti-Neutrophil Elastase antibody Abcam Cat#ab68672
InVivoMab anti-mouse PD1 (clone RMP1-14) BioXCell Cat# BE0146; RRID:AB_10949053
secondary antibodies Dako EnVision+ System- HRP Labelled Polymer Anti-Rabbit ; Agilent Code K4002
InVivoMAb anti-mouse OX40L (CD134L) (clone RM134L) Cat# BE0033
InVivoMAb anti-mouse 4-1BBL (CD137L) (clone TKS-1) Cat# BE0110

Validation

Rat anti-mouse CD3 molecular complex (clone 17A2) BD Biosciences Cat#741982
Reactivity: Mouse (Tested in Development) / Application: Flow cytometry (Qualified)
Hamster anti-mouse CD3ε (clone 145-2C11), purified BioLegend Cat#100302
Reactivity: Mouse (Tested in Development) / Application: FC - Quality tested ; IHC-F – Validated ; IP, Activ, Block, WB, ICC - Reported in the literature
Rat anti-mouse CD4 (clone GK1.5) BD Biosciences Cat#562891; RRID:AB_2737870
Reactivity: Mouse (Tested in Development) / Application: Flow cytometry (Qualified)
Rat anti-mouse CD8a (clone 53-6.7) BD Biosciences Cat#563068; RRID:AB_2687548
Reactivity: Mouse (Tested in Development) / Application: Flow cytometry (Qualified)
InVivoMab anti-mouse CD8a (clone YTS 169.4) BioXCell Cat#BE0117; RRID:AB_10950145
Reactivity: Mouse / Reported Applications: in vivo CD8+ T cell depletion, Western blot
Rat anti-CD11b (clone M1/70) BD Biosciences Cat#741934
Reactivity: Mouse (Tested in Development) / Application: Flow cytometry (Qualified)
Hamster anti-mouse CD28 (clone 37.51), purified BioLegend Cat#102102
Reactivity: Mouse / Application: FC - Quality tested; IP, IHC-F, Costim, Block - Reported in the literature
Rat anti-mouse CD45 (clone 30-F11) BD Biosciences Cat#564279; RRID:AB_2651134
Reactivity: Mouse (Tested in Development) / Application: Flow cytometry (Qualified)
Rat anti-mouse CD45R/B220 (clone RA3-6B2) BD Biosciences Cat#563892
Reactivity: Mouse (Tested in Development) / Application: Flow cytometry (Qualified)
Hamster anti-mouse CD69 (clone H1.2F3) BD Biosciences Cat#552879
Reactivity: Mouse (Tested in Development) / Application: Flow cytometry (Qualified)
Rat anti-mouse CD335 (clone 29A1.4) (αNK-p46) BD Biosciences Cat#562850
Reactivity: Mouse (Tested in Development) / Application: Flow cytometry (Qualified)
Rat anti-mouse F4/80 (clone BM8) BioLegend Cat#123114
Reactivity: Mouse (Tested in Development) / Application: Flow cytometry (Qualified)
Rabbit anti-GFP mAb (clone D5.1) (detects GFP, YFP, CFP) Cell Signaling Technology Cat#2956
REACTIVITY: All / Application: Dilution Western Blotting 1:1000 / Immunohistochemistry (Paraffin) 1:200
Mouse anti-human/mouse Granzyme B (clone QA16A02) BioLegend Cat#372204
Reactivity: Human, Mouse / Application ICFC - Quality tested
Rat anti-mouse Ly6C (clone HK1.4) BioLegend Cat#128006
Reactivity: Mouse / Application: FC (Quality tested)
Rat anti-mouse Ly6G (clone 1A8) BD Biosciences Cat#562737; RRID:AB_2737756
Reactivity: Mouse (Tested in Development) / Application: Flow cytometry (Qualified)
CD137L (4-1BBL) Antibody, anti-mouse, REAfinity (Clone: REA962) Miltenyi Biotec Cat#: 130-116-011
Reactivity: Mouse / Application FC (QC tested)
CD252 (OX40L) Antibody, anti-mouse, APC, REAfinity (Clone: REA960) Miltenyi Biotec Cat#: 130-116-073
Reactivity: Mouse / Application FC (QC tested)
InVivoMab anti-mouse Ly6G (clone 1A8) BioXCell Cat#BE0075-1; RRID:AB_1107721
Reactivity: Mouse / Reported Applications: in vivo neutrophil depletion, in vivo MDSC depletion, Immunofluorescence, Immunohistochemistry (paraffin), Immunohistochemistry (frozen), Flow cytometry
Rabbit anti-Neutrophil Elastase antibody Abcam Cat#ab68672
Species reactivity Reacts with: Mouse, Human / Tested applications Suitable for: ICC/IF, WB, IHC-Fr, IHC-P
InVivoMab anti-mouse PD1 (clone RMP1-14) BioXCell Cat# BE0146; RRID:AB_10949053
Reactivity: Mouse / Reported Applications: in vivo blocking of PD-1/PD-L signaling
InVivoMAb anti-mouse OX40L (CD134L) (clone RM134L) Cat# BE0033
Reactivity: Mouse / Reported Applications: in vivo blocking of OX-40L signaling
InVivoMAb anti-mouse 4-1BBL (CD137L) (clone TKS-1) Cat# BE0110
Reactivity: Mouse / Reported Applications: in vivo blocking of 4-1BBL signaling

Eukaryotic cell lines

Policy information about cell lines

Cell line source(s)

Mouse: B16-F10 // ATCC Cat#CRL-6475; RRID: CVCL_0159
Mouse: LL/2 (LLC1) // ATCC Cat#CRL-21642; RRID: CVCL_4358
Mouse: C57BL/6N-Atm1Brd Ppm1dtm1a(KOMP)Wtsi ES cells Knockout Mouse Project Repository CSD 44399; KOMP: CSD44399-1a-Wtsi
Human: DLD-1 colorectal adenocarcinoma cell line // ATCC CCL-221; RRID:CVCL_0248
Human: HCT 116 Colorectal carcinoma cell line // ATCC CCL-247; RRID:CVCL_0291
Human: HCT 116 Colorectal carcinoma, p53-null // Bert Vogelstein RRID:CVCL_HD97

Authentication

All experimental cell lines were obtained from ATCC with the proper certificates of authentication and used at early passages

	from unfreezing original vial. The ATCC used SRT and isoenzymes profiles for cell authentication. further authentication was not required.
Mycoplasma contamination	All cell lines were tested negative for mycoplasma contamination
Commonly misidentified lines (See ICLAC register)	No commonly misidentified cell lines were used in this study.

Animals and other organisms

Policy information about studies involving animals; ARRIVE guidelines recommended for reporting animal research

Laboratory animals	Mouse: C57BL/6J The Jackson Laboratory Cat#000664; RRID: IMSR_JAX:000664<br>Mouse: R26R-EYFP: B6.129X1-Gt(ROSA)26Sortm1(EYFP)Cos/J The Jackson Laboratory JAX:006148; RRID: IMSR_JAX:006148<br>Mouse: LysM-cre: B6.129P2-Lyz2tm1(cre)Ifo/J The Jackson Laboratory JAX:004781; RRID: IMSR_JAX:004781<br>Mouse: MRP8-creTg: B6.Cg-Tg(S100A8-cre,-EGFP)1Ilw/J The Jackson Laboratory JAX:021614; RRID: IMSR_JAX:021614<br>Mouse: β-actin-creTg: C57BL/6J-Tg(β-actin-cre) Lewandowski et al. 1997 MGI:J:46833<br>Mouse: β-actin-flpTg: C57BL/6J-Tg(β-actin-flp) Dymecki et al. 1996 MGI:2158498<br>Mouse: FES-creTg: C57BL6-Tg(FES-cre)31Bsl Keller et al. 2001 MGI:2450256<br>Mouse: Trp53KO:B6.129-Trp53tm1Brd/N Donehower et al. 1992 MGI:1857590<br>Mouse: UBC-Ppm1dTg: C57BL6-Tg(UBC-Ppm1d) Wong et al. 2009 N/A<br>Mouse: Ppm1dKO2:C57BL6-Ppm1dTm1b(KOMP)Wtsi This manuscript N/A<br>Mouse: Ppm1dfl: C57BL6-Ppm1dTm1c(KOMP)Wtsi This manuscript N/A<br>Mouse: Ppm1dKO3: C57BL6-Ppm1dTm1d(KOMP)Wtsi This manuscript N/A<br>All animals were bred and maintained in specific pathogen-free facilities with 12 light/12 dark cycle, temperature ~20-23°C, 45-60% humidity. Animals had water ad libitum and were fed regular chow. Experiments were performed on 8 to 12-week-old female and males of the immunocompetent C57Bl/6 background. 2 to 12-months-old female and males were used in experiments for Figure 1a. Littermate animals from different cages were randomly assigned into experimental groups and were either co-housed or systematically exposed to other groups' bedding to ensure equal exposure to common microbiota.
Wild animals	No wild animals were used in the study.
Field-collected samples	No field-collected samples were used.
Ethics oversight	All experiments were carried out in accordance with FELASA and Animal Experimental Ethics Committee guidelines (University of Burgundy, France) or with NCI Animal Care and Use Committee guidelines (NCI).

Note that full information on the approval of the study protocol must also be provided in the manuscript.

Human research participants

Policy information about studies involving human research participants

Population characteristics	The population characteristics were provided in Supplementary table 1 for human tumor samples (4- females , 1- male; age range 53-81). Human neutrophils and T cells were isolated from the blood of adult healthy donors randomly supplied from Etablissement Français du Sang Bourgogne Franche-Comté, Besançon, France.
Recruitment	Five patients with stage I–II lung cancer, who were scheduled for surgical resection, consented to tissue collection of a portion of their tumor and/or blood for research purposes Institutional Review Board of the Hospital of the University of Pennsylvania. There has been no potential self-selection bias because lung cancer patients and blood donors were selected randomly. Buffy coats from random donors were purchased from The French Blood Bank (EFS) – Bourgogne Franche-Comté.
Ethics oversight	This study was approved by the Institutional Review Board of the Hospital of the University of Pennsylvania. For donors's blood, ethical review and approval were done by the French Blood Transfusion Center (Etablissement Français du Sang Bourgogne Franche-Comté, Besançon, France).

Note that full information on the approval of the study protocol must also be provided in the manuscript.

Flow Cytometry

Plots

Confirm that:

- [x] The axis labels state the marker and fluorochrome used (e.g. CD4-FITC).
- [x] The axis scales are clearly visible. Include numbers along axes only for bottom left plot of group (a 'group' is an analysis of identical markers).
- [x] All plots are contour plots with outliers or pseudocolor plots.
- [x] A numerical value for number of cells or percentage (with statistics) is provided.

Methodology

Sample preparation	Single-cell suspensions were re-suspended in BD stain buffer (BD Bioscience) for 15 min prior to staining with specific antibodies. Antibodies against cell surface markers: anti-CD45, anti-CD3, anti-CD4, anti-CD8a, anti-CD11b, anti-F4/80, anti-Ly6G, anti-Ly6C, anti-NKp46, anti-B220, anti-CD69, were purchased from BD Bioscience and BioLegend (detailed in Key Resources Table). Samples were mixed with FVS700 (1/7000) and data were acquired on an LSR Fortessa flow cytometer (BD Biosciences) and analyzed with FlowJo software (Tree Star). ROS generation in cultured neutrophils was determined using a dihydroethidium (DHE) fluorescent probe. Cells were incubated with DHE (10 μM) in HBSS containing 1.5 mM CaCl2 and 1mM MgCl2 for 30 min at 37°C and analyzed by flow cytometry. LacZ activity was determined by flow cytometry using Fluorescein di[β-D-galactopyranoside] (Sigma, F2756) as described elsewhere (see ref 91)
Instrument	Flow cytometry data was collected on a BD LSR FORTESSA. Flow cytometry sorting was performed on a BD FACSAria-III (BD Biosciences).
Software	Flow cytometry data were analyzed on FlowJo Tree Star (V10.0.2)
Cell population abundance	Human CD3+ T cells were obtained from buffy coat preparations of human healthy donor blood. T cells were urified using a Pan T Cell Isolation Kit and re-stimulated with a human T Cell Activation/Expansion Kit (Miltenyi Biotec).<br>Mouse Naive CD3+ T cells were obtained from spleens and lymph nodes of C57BL/6 wild-type mice. Cells were purified using the MACS Cell Separation system (Pan T Cell isolation kit, Miltenyi Biotec).<br>Mouse neutrophils were obtained using a mouse Neutrophil Isolation Kit (Miltenyi Biotec).<br>TANs were isolated from tumor single cell suspensions using positive selection of CD66b+ cells with microbeads as previously described (Eruslanov JCI 2014, Singhal Cancer Cell 2016).<br>PBNs were obtained from EDTA anti-coagulated peripheral blood collected from lung cancer patients during surgery or from healthy donors. The PBNs were obtained from Lymphoprep (Accu-Prep, 1.077 g/ml, Oslo, Norway) density gradient centrifugation followed by erythrocyte lysis with 1x RBC Lysis Buffer.<br>Purity of isolated cells was confirmed by cytometry and cells were pure at >90%
Gating strategy	Tumor infiltrating cells were selected using FSC/SSC gating, CD45+ live cells were selected (based on viability staining), and different cell population were analysed in Live CD45+ cells, using FMO samples as control. Gating strategies are presented in Extended data Fig. S3.

☒ Tick this box to confirm that a figure exemplifying the gating strategy is provided in the Supplementary Information.
